# Two MYB transcription factors (CsMYB2 and CsMYB26) are involved in flavonoid biosynthesis in tea plant [*Camellia sinensis* (L.) O. Kuntze]

**DOI:** 10.1186/s12870-018-1502-3

**Published:** 2018-11-20

**Authors:** Wen-Li Wang, Yong-Xin Wang, Hui Li, Zhi-Wei Liu, Xin Cui, Jing Zhuang

**Affiliations:** Tea Science Research Institute, College of Horticulture, Nanjing Agricultural University, 1 Weigang, Nanjing, 210095 Jiangsu China

**Keywords:** Tea plant, Flavonoid biosynthesis, CsMYB2, CsMYB26, Subcellular localization, Transcriptional level

## Abstract

**Background:**

Flavonoids are secondary metabolites that play important roles in the entire tea plant life cycle and have potential health-promoting properties. MYB transcription factors (TFs) are considered potentially important regulators of flavonoid biosynthesis in plants. However, the molecular mechanisms by which MYB TFs regulate the flavonoid pathway in tea plant remain unknown.

**Results:**

In this study, two R2R3-MYB TFs (CsMYB2 and CsMYB26) involved in flavonoid biosynthesis in tea plant were investigated. The genes encoding CsMYB2 and CsMYB26 were cloned from the tea plant cultivar ‘Longjing 43’. Phylogenetic analysis showed that CsMYB2 and CsMYB26 were grouped into the proanthocyanidin biosynthesis-related MYB clade. Multiple sequence alignment revealed that conserved motif 1 in the two MYB factors was related to the bHLH TF. Subcellular localization assays suggested that CsMYB2 localized in the nucleus. Promoter analysis indicated that *CsMYB2*, *CsMYB26* and the related structural genes contain MYB recognition elements. The expression levels of the *CsMYB2* and *CsMYB26* genes and the structural genes in the flavonoid biosynthesis pathway were determined in leaves from various sites in the two tea plant cultivars ‘Longjing 43’ and ‘Baiye 1 hao’.

**Conclusions:**

The expression levels of these genes were correlated with the accumulated flavonoid content. The results demonstrated that the expression level of *CsF3’H* may be regulated by CsMYB2 and that *CsMYB26* expression is positively correlated with *CsLAR* expression. The relative transcriptional level of *CsMYB26* may be the main reason for the different epigallocatechin contents between the tea plant cultivars ‘Longjing 43’ and ‘Baiye 1 hao’. Our results will serve as a reference for the potential regulatory roles of CsMYB2 and CsMYB26 in flavonoid biosynthesis in tea plant and may also assist biologists in improving tea quality.

**Electronic supplementary material:**

The online version of this article (10.1186/s12870-018-1502-3) contains supplementary material, which is available to authorized users.

## Background

Tea plant [*Camellia sinensis* (L.) O. Kuntze] is an important economic leaf crop worldwide, and tea is known as a healthy beverage [1]. In tea plants, catechins, anthocyanidins, and proanthocyanidins (PAs) are important secondary metabolites that are synthesized via the flavonoid pathway.

As one of the most researched secondary metabolism pathways in tea plant growth processes, the flavonoid pathway is divided into the “early” flavonoid pathway and the “late” flavonoid pathway [2]. In the “early” flavonoid pathway, the synthesis of different metabolites shares the same structural genes and biosynthetic precursors, namely, chalcone synthase (*CHS*), chalcone isomerase (*CHI*), flavanone 3-hydroxylase (*F3H*), flavanone 3′-hydroxylase (*F3’H*), and flavanone 3′,5′-hydroxylase (*F3’5’H*). Correspondingly, the biosynthesis of the different secondary metabolites catechin, anthocyanin, and PA is mainly controlled by the “late” flavonoid pathway. For example, the leucoanthocyanidin reductase (*LAR*) gene is key for the production of catechin (*C*); anthocyanidin synthase (*ANS*) and anthocyanidin reductase (*ANR*) are important structural genes for the production of epicatechin (EC), gallocatechin (GC), epigallocatechin (EGC), epicatechin gallate, and epigallocatechin gallate. Different catechin monomers are substrates for UDP-glucose:flavonoid 3-O-glucosyltransferase (*UFGT*) in the production of anthocyanins and the hydroxylation and polymerization of flavan-3-ol to form PAs [3, 4].

In higher plants, flavonoid biosynthesis is not only regulated by structural genes but also involves a number of regulatory genes [5, 6]. Studies have demonstrated that different transcription factor (TF) families, such as the MYB, bHLH, and WD40 families, physically interact to form the MBW complex and are responsible for regulating flavonoid biosynthesis [6, 7].

The MYB family is one of the largest TF families in plants. The R2R3-MYB-type family contains the largest number of members. In *Arabidopsis thaliana*, according to functional analysis, AtR2R3-MYB-type family members are divided into 25 subgroups [8, 9]. Members of the MYB 5 subgroup mainly control PA metabolism in the flavonoid pathway; however, to date, only AtMYB123 has been found to belong in this subgroup in *A. thaliana* [10]. In the R2R3-MYB-type TF family, the conserved motif (DNEI[A/S/G]N[D/A/N]V) was proven to bind to a specific site in BHLH. Moreover, this motif has been found in the R3 structural domain [9, 11]. The N-terminal MYB domains are very conserved, in the N-terminal but those in the C-terminal vary. The C-terminal often contains transcriptional activation or repression domains as well as conserved serine and threonine residues, which may correspond to posttranslational modification sites [12].

The sequence features of R2R3-MYB TFs and the regulatory activities of these TFs in the flavonoid pathway have been investigated in various plants [13–17]. Nathalie et al. found that *TT2* at least partially determined PA accumulation [10]. The overexpression of *VvMYBPA1* in *Arabidopsis* induced the upregulation of flavonoid pathway genes and the accumulation of PAs [18]. Laurent et al. elucidated the ability of the *VvMYB5a* gene to be mainly expressed during the early stages of berry development in skin, flesh, and seeds. The overexpression of *VvMYB5a* in tobacco affected the expression profiles of structural genes controlling the synthesis of phenylpropanoids and the metabolism of anthocyanins [19]. There is a positive linear correlation between *PpMYB10* and *PpMYBPA1* and the metabolites of the flavonoid pathway. Ravaglia et al. concluded that anthocyanins are regulated by MYB10 and that PAs are regulated by MYBPA1 [20]. Xu et al. found that *DcMYB6* TFs are involved in regulating anthocyanin biosynthesis in purple carrot taproots [6]. An R2R3-MYB TF, OjMYB1, was reported to be involved in anthocyanin biosynthesis in *Oenanthe javanica* [5]. AgMYB2 is involved in the regulation of anthocyanin biosynthesis in purple celery [21]. *MdMYBA* induces anthocyanin accumulation in the reproductive tissues of transgenic tobacco [22]. *CsMYB4a* is potentially involved in the regulation of flavonoid gene expression [23]. *CsAN1* was isolated from the ‘Zijuan’ cultivar and found to regulate anthocyanin accumulation [13].

R2R3 MYB TFs are essential for plants and are believed to play important roles in regulating the flavonoid pathway in various species [6, 13, 24–26]. However, the molecular mechanisms by which MYB TFs regulate the flavonoid pathway in tea plant remain unknown. In the present study, we investigated two MYB TFs (CsMYB2 and CsMYB26) involved in the flavonoid metabolism pathway in tea plant. In addition, we collected leaves from different sites on two tea plant cultivars, ‘Longjing 43’ and ‘Baiye 1 hao’. The former cultivar is a stable early budding cultivar, while the latter is a typical low temperature-sensitive small leaf cultivar. Different metabolites (catechins, anthocyanins, and PAs) in the flavonoid pathway were detected. Our results will provide a reference on how MYB TFs regulate the flavonoid pathway in tea plant and may also assist biologists in improving tea plant quality.

## Results

### Cloning of *CsMYB2* and *CsMYB26* genes from tea plant

**Fig. 1:**
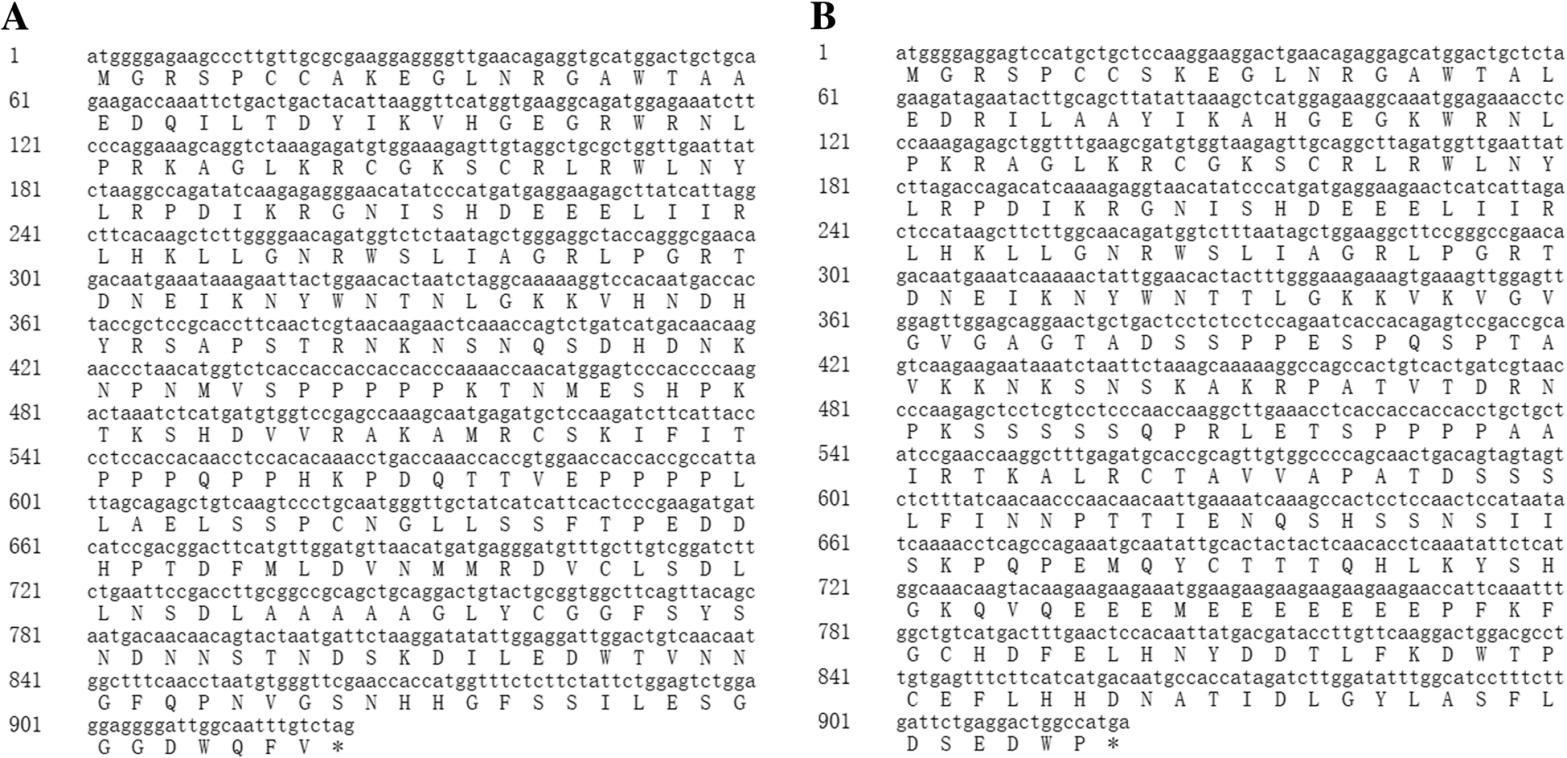
The two tea plant cultivars. **a** ‘Longjing 43’ plant. **b** ‘Baiye 1 hao’ plant

The *CsMYB2* and *CsMYB26* genes were cloned from the tea plant cultivar ‘Longjing 43’. The amplification products and open reading frames (ORFs) of the cloned sequences comprised 924 and 921 bp, respectively. The integral nucleotide sequences and deduced amino acid sequences are shown in Fig. 1.

### Phylogenetic tree analysis and conserved motif analysis of the CsMYB2 and CsMYB26 TFs

**Fig. 2:**
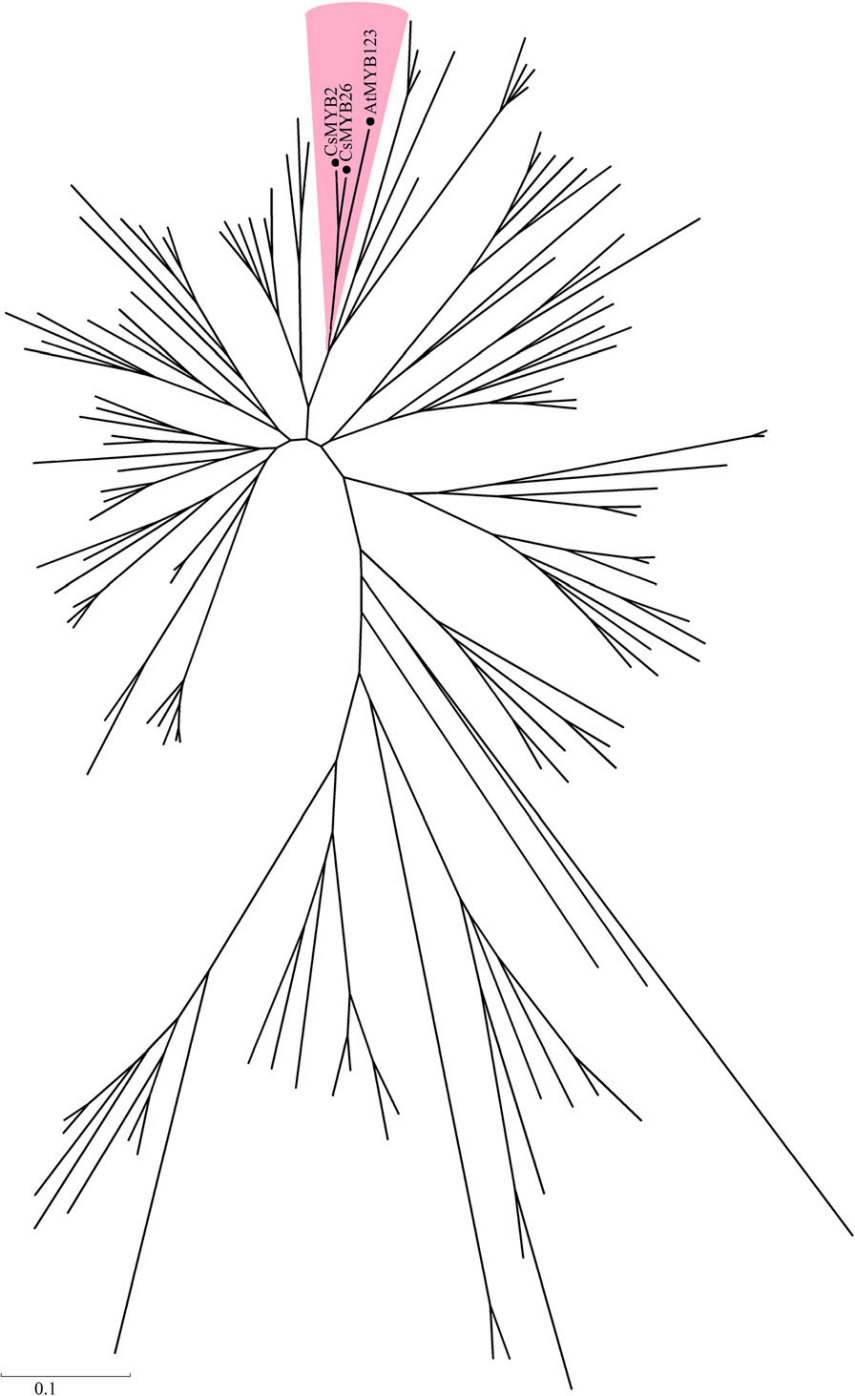
Gene sequences of *CsMYB2* and *CsMYB26* with the deduced amino acid sequences. **a** *CsMYB2* gene. **b** *CsMYB26* gene

**Fig. 3:**
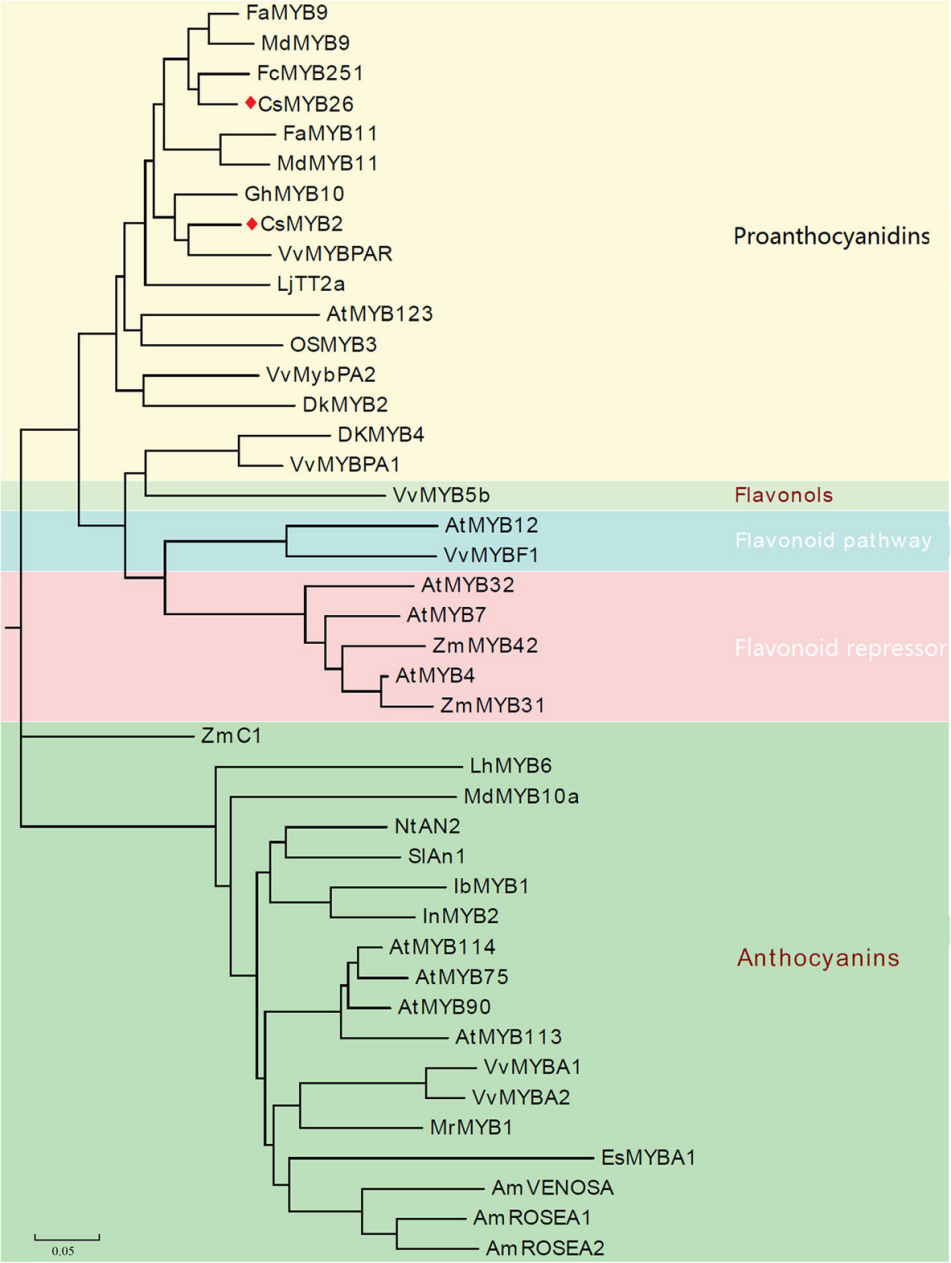
Unrooted phylogenetic tree of CsMYB2 and CsMYB26 with R2R3-MYB-type *A. thaliana* TFs. A phylogenetic tree was built using the neighbor-joining method with MEGA 5 software. The putative functions of all R2R3-MYBs are listed on the right

To analyse the evolutionary relationships of CsMYB2 and CsMYB26 in tea plant with those in *Arabidopsis*, an unrooted phylogenetic tree was constructed. As shown in Fig. 2, CsMYB2 and CsMYB26 were classified into subgroup 5. CsMYB2 and CsMYB26 were closely related to AtMYB123, which is involved in the flavonoid pathway. The amino acid sequences of homologs involved in the flavonoid pathway from various species, including FaMYB9 and FaMYB11 from strawberry (*Fragaria ananassa*); MdMYB9 and MdMYB11 from apple (*Malus domestica*); FcMYB251 from Japanese beech (*Fagus crenata*); VvMYBPAR, VvMYBPA2, and VvMYBPA1 from grapevine (*V. vinifera*); LjTT2a from lotus (*Lotus japonicas*); AtMYB123 from *Arabidopsis*; OsMYB3 from rice (*Oryza sativa*); and DkMYB2 and DkMYB4 from persimmon (*Diospyros kaki*), were selected to construct the phylogenetic tree. The phylogenetic analysis indicated that CsMYB2 and CsMYB26 have markedly orthologous relationships with VvMYBPAR and FcMYB251, respectively. CsMYB2 and CsMYB26 were grouped into the PA biosynthesis-related MYB clade (Fig. 3).

**Fig. 4:**
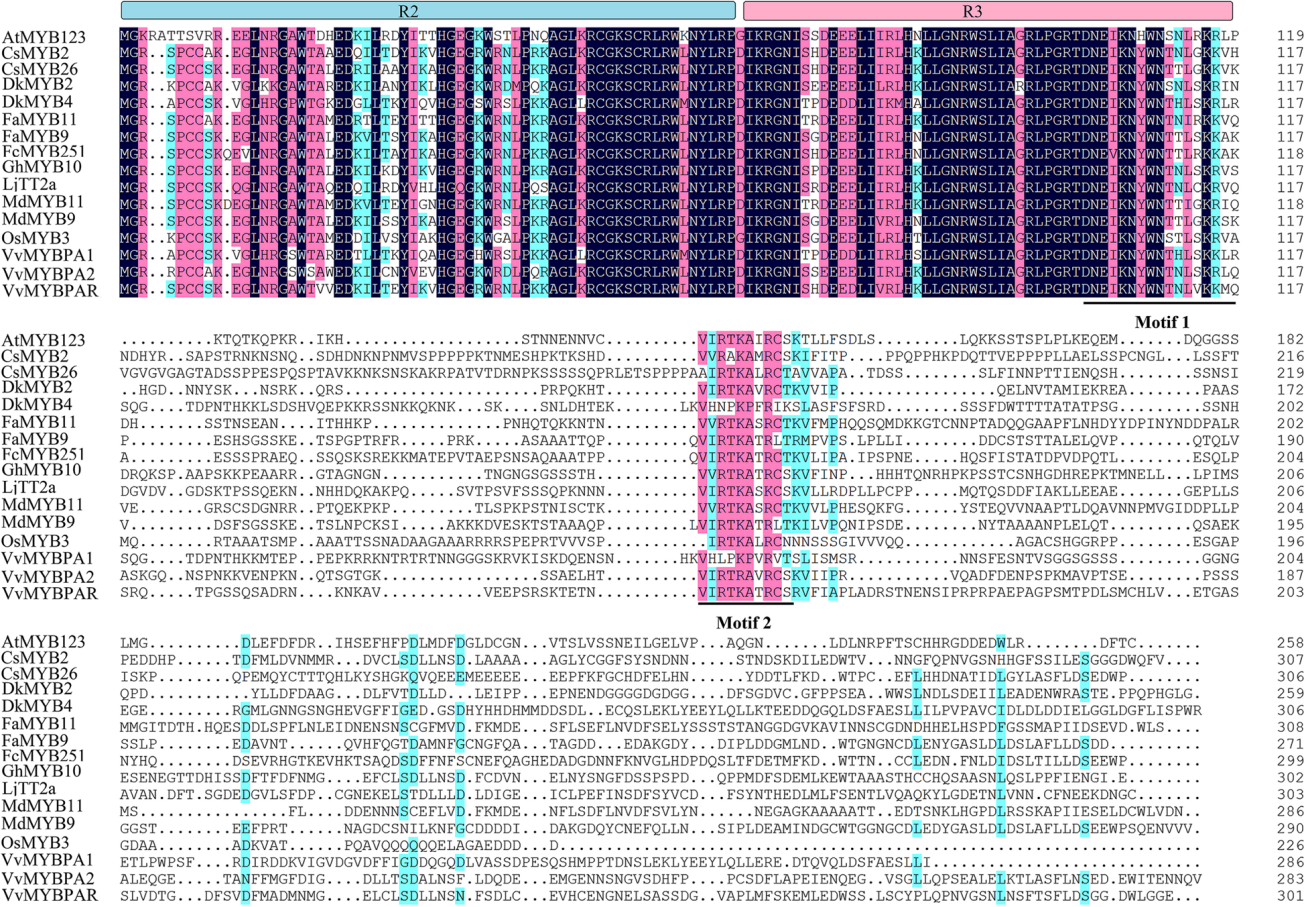
Phylogenetic relationships among CsMYB2, CsMYB26 and flavonoid-related R2R3-MYBs from other plant species. A phylogenetic tree was built using the neighbor-joining method with MEGA 5 software. The putative functions of all R2R3-MYBs are listed on the right

The TFs involved in the PA pathway, along with the CsMYB TFs were selected, and multiple sequence alignment was performed. Figure 4 shows that the R2 and R3 domains were conserved in these species and that different sequences of motif1 and motif2 from the same subgroup were highly conserved. Moreover, motif1 is also known as the bHLH motif, which is required for the interaction with the bHLH protein.

### Subcellular localization analysis of CsMYB2

**Fig. 5:**
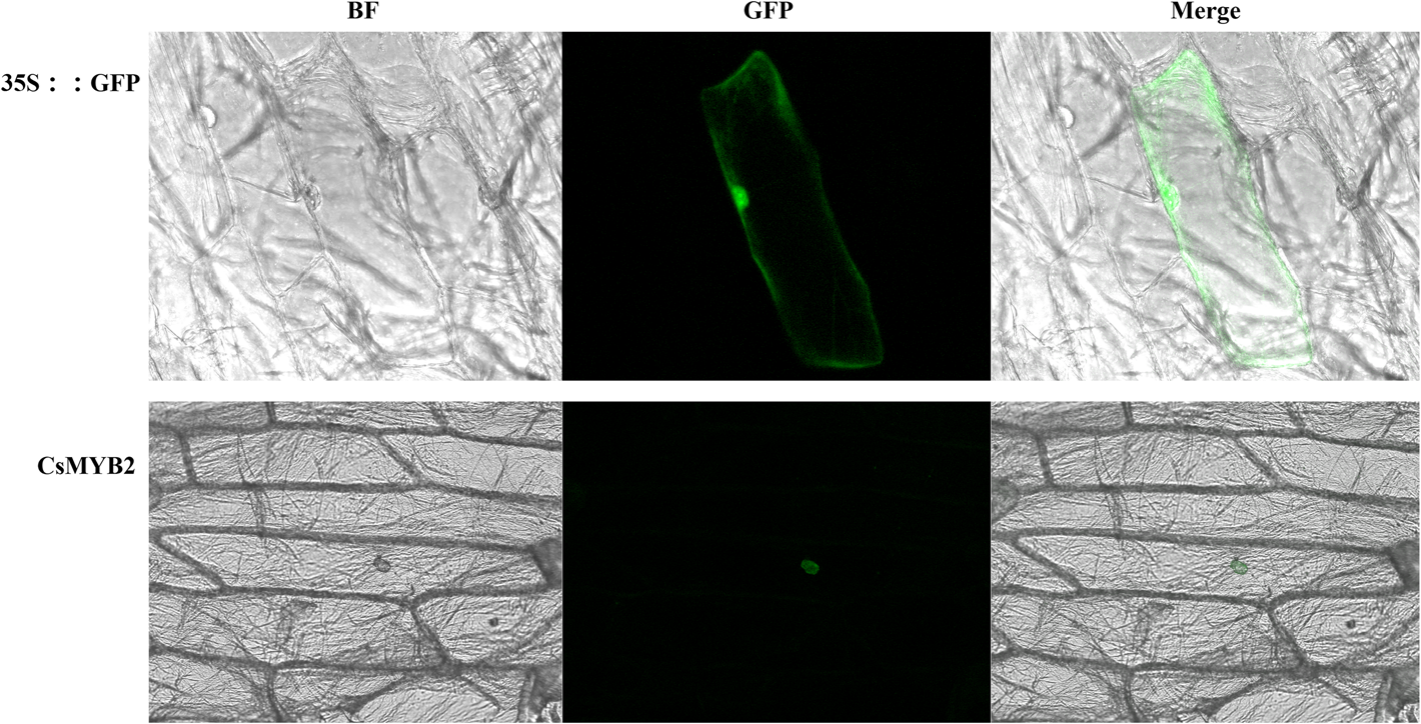
Alignment of the deduced amino acid sequences of CsMYB2 and CsMYB26 with those of R2R3-MYB proteins from other plant species

The ORF of the *CsMYB2* gene was inserted into the GFP reporter gene under the control of the CaMV 35S promoter. The *CsMYB2-GFP* fusion gene and *GFP* recombinant constructs, as controls, were introduced into onion epidermal cells by particle bombardment. The results showed that the CsMYB2-GFP fusion protein was specifically localized in the nucleus (Fig. 5). The GFP signal from the empty vector showed ubiquitous distribution throughout the cell.

### Interaction network of CsMYB2, CsMYB26 and the structural genes involved in the flavonoid pathway in tea plant

**Fig. 6:**
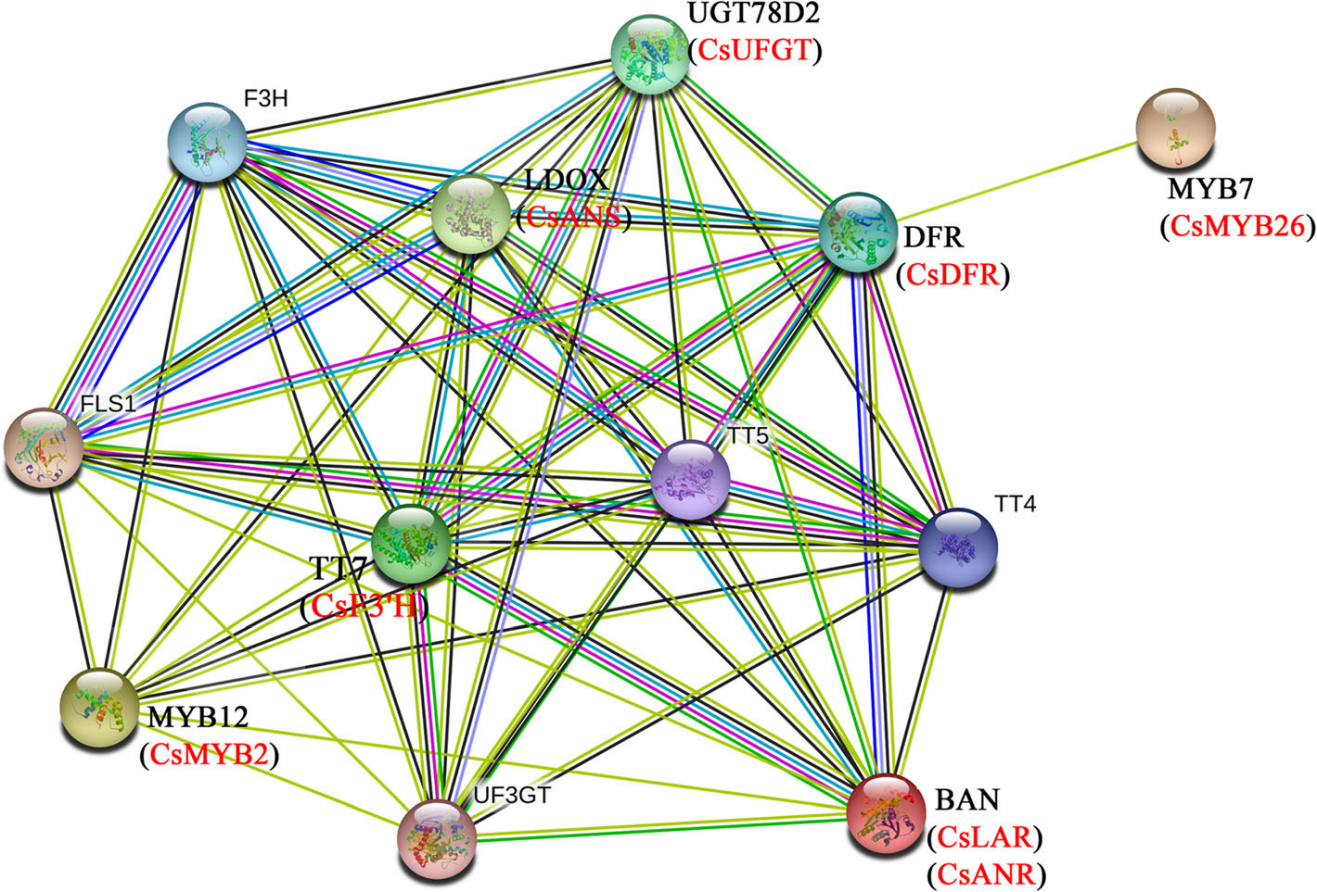
Subcellular localization of CsMYB2. BF: Bright-field microscopy image. GFP: Green fluorescence image. Merge: Merged bright-field and green fluorescence images

To better understand the interactions among CsMYB2, CsMYB26 and the structural genes involved in the flavonoid pathway in tea plant, an interaction network was built using STRING software on the basis of the orthologs in *Arabidopsis* (Fig. 6). MYB12 (CsMYB2) is a flavonol-specific activator of flavonoid biosynthesis that can activate the expression of CHS, CHI, F3H, and FLS1. MYB12 (CsMYB2) interacts with BAN (CsANR and CsLAR), UGT78D2 (CsUFGT), TT7 (CsF3’H), DFR (CsDFR), and LDOX (CsANS), while MYB2 (CsMYB26) only interacts with DFR (CsDFR).

### Analysis of the promoter regions of *CsMYB2*, *CsMYB26* and the structural genes involved in the flavonoid pathway in tea plant

The sequences of *CsMYB2*, *CsMYB26* and the related structural genes (*CsF3’H*, *CsDFR*, *CsANS*, *CsANR*, *CsLAR*, and *CsUFGT*) 2000 bp upstream of the transcription start site were analysed to understand the regulatory mechanisms that control the expression of these genes. As shown in (Additional file 1), several regulatory elements, which are related to important physiological processes, such as the light response, hormonal/environment responses, and developmental regulation, were found in the promoter regions. Several light-responsive elements were widely present in the promoter regions, including Box4, ACE, G-box, GT1 motif, and TCT motif elements. This finding suggested that the expression of these genes might be related to photosynthesis and carbohydrate metabolism. Moreover, *cis*-regulatory elements were present in the promoter regions of *CsMYB2*, *CsMYB26*, *CsF3’H*, *CsDFR*, *CsANS*, *CsANR*, *CsLAR*, and *CsUFGT.* These elements are related to the signaling pathways of abscisic acid (ABA), ethylene (ERE), methyl jasmonic acid (MeJA), jasmonic acid (SA), and auxin. Among these elements, ABA, SA, and MeJA are important signaling molecules in plant responses to stress, indicating that most of these genes are involved in responses to biotic and abiotic stresses. The promoters of these genes contain many MBS, ARE, W box, STRE, and TC-rich repeat elements, indicating that these genes are subject to stress regulation. Additionally, the promoters of these genes contain many MYB recognition site elements. Among these elements, MYB recognition sites were found in all genes examined. The results suggested that the expression of structural genes might be regulated by *CsMYB* genes and that *CsMYB2* and *CsMYB26* might also be regulated by other *CsMYB* genes.

### Expression profiles of *CsMYB2*, *CsMYB26* and the structural genes under ABA and shading treatments

**Fig. 7:**
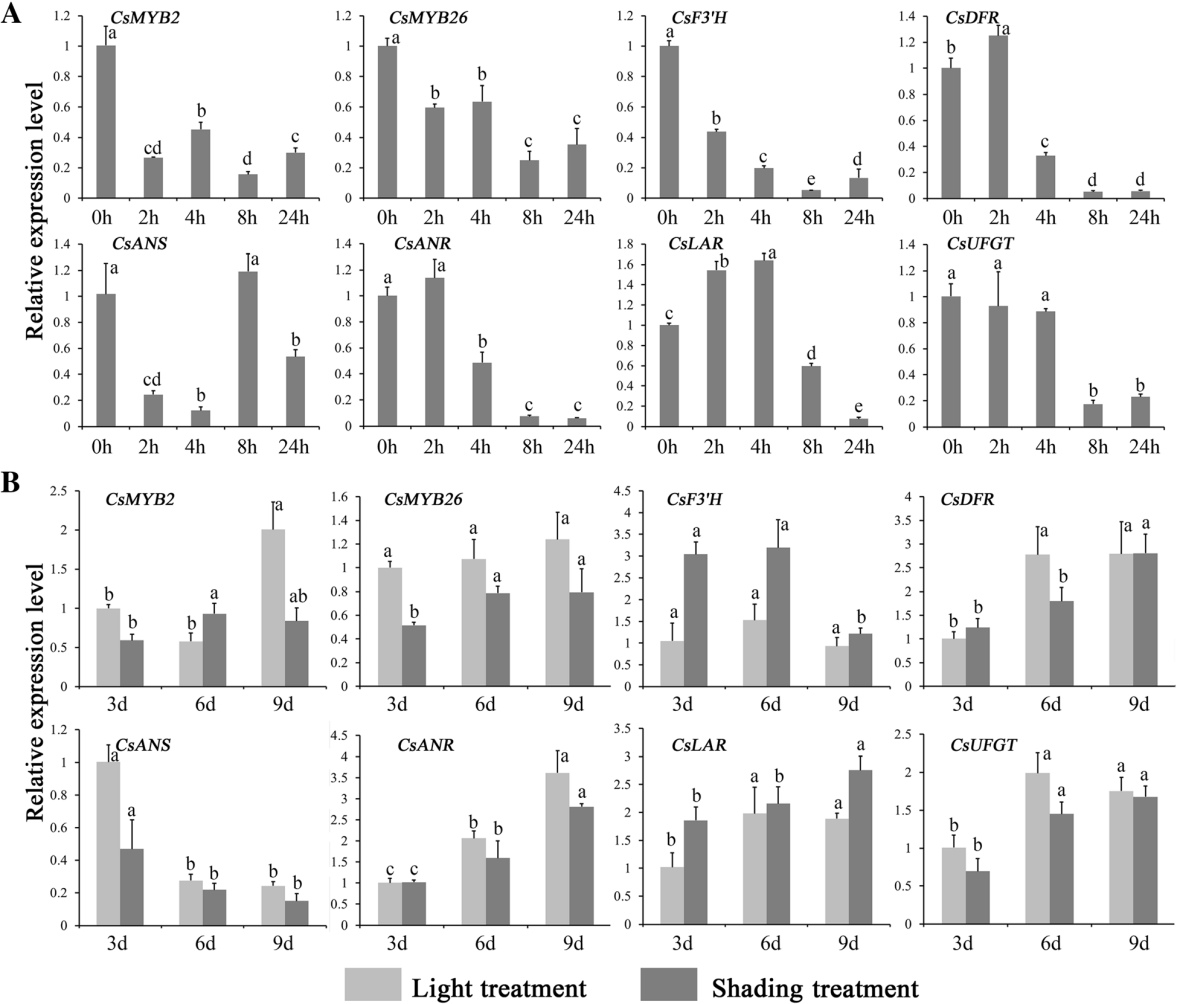
Interaction network of CsMYB2, CsMYB26 and the structural genes involved in flavonoid biosynthesis

The expression levels of *CsMYB2* and *CsMYB26* along with those of the structural genes involved in the flavonoid pathway (*CsF3’H*, *CsDFR*, *CsANS*, *CsANR*, *CsLAR*, and *CsUFGT*) were determined under ABA and shading treatments (Fig. 7).

#### ABA treatment

As shown in Fig. 7a, almost all tested genes were gradually downregulated. However, *CsLAR* was first upregulated at 2 and 4 h and was then gradually downregulated.

#### Shading treatment

The expression levels of most tested genes were increased with increasing time in both sunlight and shade conditions (Fig. 7b). The expression levels of *CsMYB2*, *CsMYB26*, *CsDFR*, *CsANS*, *CsANR*, and *CsUFGT* were lower under shading treatment than under sunlight treatment. Conversely, *CsF3’H* and *CsLAR* exhibited higher expression levels under shading treatment than under sunlight treatment.

### Relationships among the expression profiles of *CsMYB2*, *CsMYB26* and the structural genes involved in the flavonoid pathway

**Fig. 8:**
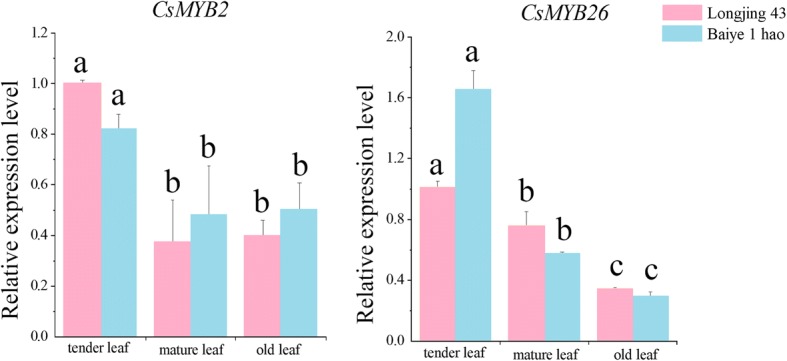
Expression profiles of *CsMYB2*, *CsMYB26* and structural genes under ABA and shading treatments. **a** ABA treatment. **b** shading treatment

As shown in Fig. 8, the expression levels of the *CsMYB2* gene for leaves from different sites on the plants were not significantly different between ‘Longjing 43’ and ‘Baiye 1 hao’. The successive decreasing order of the expression levels was tender leaves > older leaves > mature leaves. In addition, mature leaves and older leaves showed no significant difference in gene expression profiles. The *CsMYB26* gene showed the lowest expression levels in old leaves and the highest expression levels in tender leaves.

**Fig. 9:**
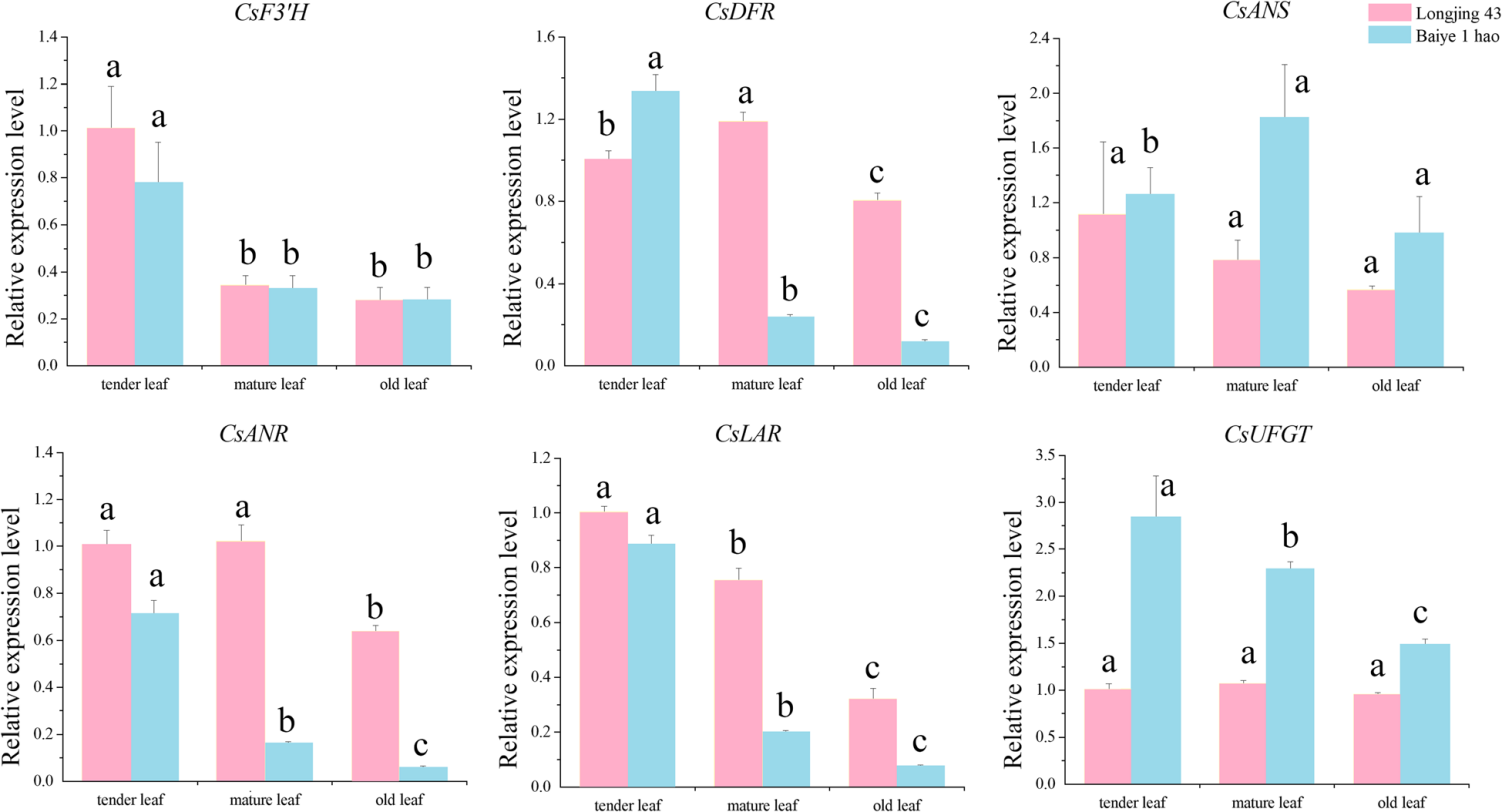
Relative expression analyses of *CsMYB2* and *CsMYB26* in the leaves from different sites in tea plant

The expression levels of genes encoding the enzymes in the flavonoid pathway (*CsF3’H*, *CsDFR*, *CsANS*, *CsANR*, *CsLAR*, and *CsUFGT*) were determined in the leaves at different sites (Fig. 9). The expression profile of *CsF3’H* was in accordance with that of *CsMYB2.* In addition, the gene expression levels in ‘Longjing 43’ were higher than those in ‘Baiye 1 hao’. In ‘Baiye 1 hao’, the expression profiles of *CsDFR*, *CsANS*, and *CsLAR* were in accordance with the *CsMYB26* expression levels*.* The highest expression levels were found in tender leaves, and the lowest expression levels were found in older leaves. However, this consistent pattern was not found in ‘Longjing 43’. In ‘Longjing 43’, *CsDFR* and *CsANR* had the highest expression profiles in mature leaves and tender leaves, with lower expression levels in tender leaves and mature leaves. The difference between the two tea plant cultivars suggested that the gene expression patterns may be due to cultivar specificity.

### Catechin (GC, EGC, C, and EC) contents in leaves from different sites in tea plant

**Fig. 10:**
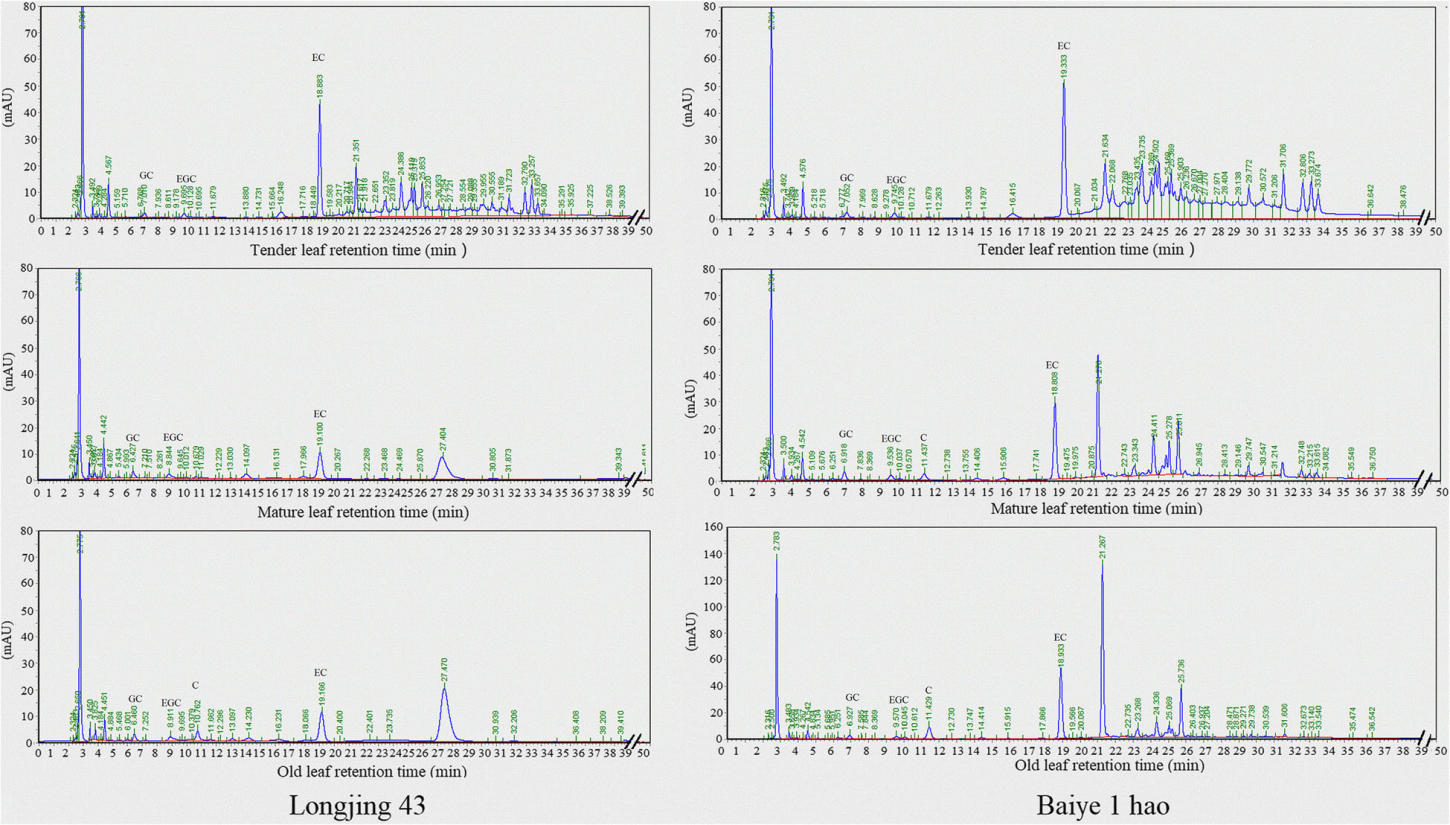
Relative expression analyses of genes involved in the flavonoid biosynthesis pathway in the leaves from different sites in tea plant

**Fig. 11:**
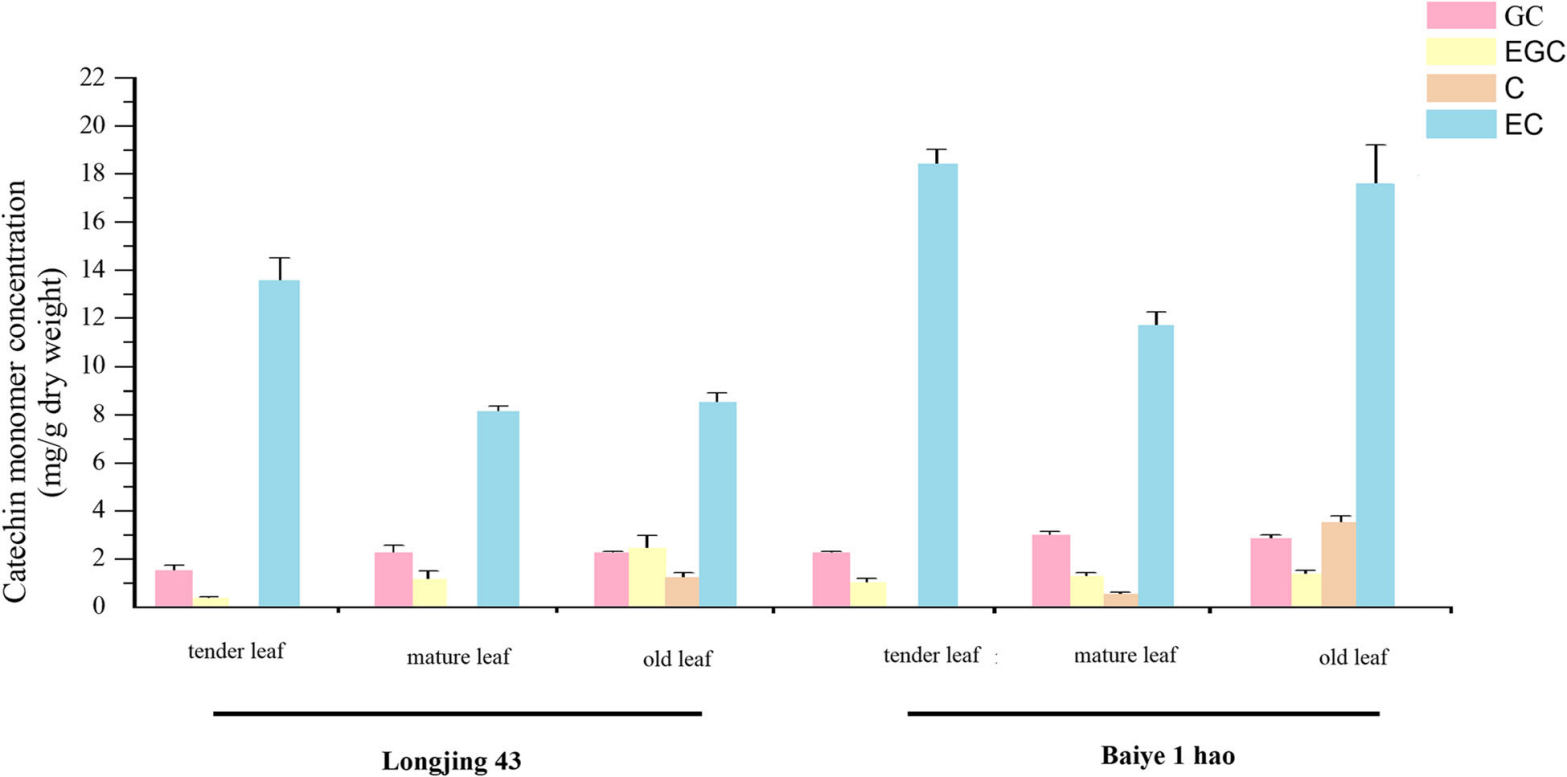
HPLC chromatogram of catechins in leaves from different sites in the ‘Longjing 43’ and ‘Baiye 1 hao’ cultivars

The contents of various monomeric catechins were detected in the ‘Longjing 43’ and ‘Baiye 1 hao’ cultivars (Figs. 10 and 11). The content of EC was much higher than that of other components, and the highest content was found in tender leaves followed by old leaves and mature leaves. In addition, the EC content in ‘Baiye 1 hao’ was higher than that in ‘Longjing 43’. The GC content in mature leaves was higher than that in tender leaves. The EGC content decreased in the following order: old leaf > mature leaf > tender leaf, especially in the ‘Longjing 43’ cultivar. The C monomer was not detected in tender leaves.

### Anthocyanidin and PA contents in the leaves from different sites in tea plant

**Fig. 12:**
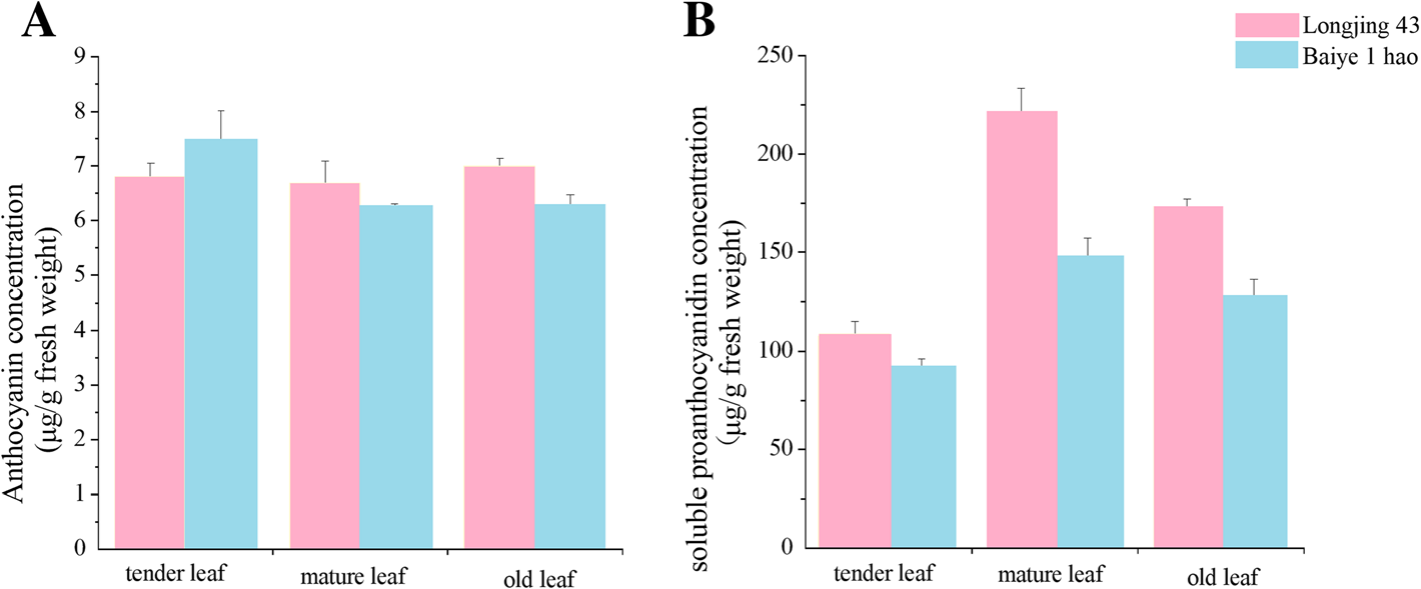
Various catechin monomer content analyses were performed

The content of anthocyanidins and PAs was also detected in the leaves from different sites in the two tea plant cultivars. The anthocyanidin contents were similar in the leaves from different sites of the two tea plant cultivars (Fig. 12a). As shown in Fig. 12b, the content of soluble PAs in the leaves from different sites coincided in the two tea plant cultivars, with the following order: mature leaves > old leaves > tender leaves.

### Correlation analysis of *CsMYB2* and *CsMYB26* levels with those of different structural genes and metabolites

The correlation coefficients were analysed by correlating *CsMYB2* and *CsMYB26* expression with that of several structural genes in the flavonoid pathway (Table 1). The results indicated that the correlation coefficients for the expression levels among the *CsMYB26*, *CsLAR*, and *CsANR* genes were significant. The expression levels of *CsMYB26* may thus be positively correlated with those of *CsLAR* and *CsANR*. In addition, the expression profiles of *CsMYB2* were notably correlated with those of *CsF3’H* in the two tea plant cultivars. The correlation coefficients of the expression profiles of *CsMYB2* and *CsMYB26* with the expression profiles of *CsDFR* was at least 0.99 in ‘Baiye 1 hao’. Therefore, we speculated that *CsMYB2* and *CsMYB26* play a potential role in the regulation of *CsDFR* expression.

**Table 1:** Correlation of expression levels between regulated genes and related structural genes based on Pearson’s correlation analysis

		Correlation coefficient					
		*CsDFR*	*CsLAR*	*CsANR*	*CsANS*	*CsUFGT*	*CsF3’H*
*CsMYB2*	Longjing 43	−0.008	0.756	0.432	0.905	−0.086	0.994
	Baiye 1 hao	0.99	0.982	0.98	−0.242	0.78	0.99
*CsMYB26*	Longjing 43	0.642	1.000^b^	0.914	0.965	0.58	0.828
	Baiye 1 hao	0.994	0.998^a^	0.999^a^	0.003	0.91	0.994

^a^Significant correlation at the 0.05 level (two-tailed)

^b^Significant correlation at the 0.01 level (two-tailed)

Correlative analyses between the concentrations of flavonoid compounds (catechins, anthocyanins, and PAs) and the expression levels of *CsMYB2* and *CsMYB26* indicated that the two MYB genes perform differently than those of the flavonoid metabolites. CsMYB2 expression was negatively correlated with GC content and positively correlated with EC content (Table 2). Moreover, CsMYB2 expression was positively correlated with anthocyanin content and negatively correlated with soluble PA content. CsMYB2 positively regulated the content of anthocyanins and negatively regulated the content of PAs in tea plant leaves. CsMYB26 was positively correlated with EGC content and might also regulate anthocyanin content in ‘Baiye 1 hao’.

**Table 2:** Catechin monomers, anthocyanins, and Pas and the expression levels of regulated genes based on Pearson’s correlation analysis

		Correlation coefficient					
		GC	EGC	C	EC	Anthocyanins	PAs
*CsMYB2*	Longjing 43	−1.000^a^	− 0.77	−0.471	0.999^a^	− 0.101	−0.912
	Baiye 1 hao	−0.949	−0.964	− 0.575	0.628	0.999^a^	− 0.952
*CsMYB26*	Longjing 43	−0.781	−1.000^b^	− 0.93	0.739	−0.722	− 0.429
	Baiye 1 hao	−0.843	−1.000^a^	− 0.758	0.418	0.98	−0.849

- undetected

^a^Significant correlation at the 0.05 level (two-tailed)

^b^Significant correlation at the 0.01 level (two-tailed)

According to the association analysis, *CsMYB26* positively regulated *CsDRF* and *CsLAR* expression. *CsMYB26* also positively regulated *CsANR* and *CsF3’H* expression in ‘Baiye 1 hao’. Moreover, *CsMYB26* expression was negatively correlated with the content of the secondary metabolite EGC. *CsMYB2* expression was positively correlated with the expression of *CsDFR* and *CsF3’H*. *CsMYB2* negatively regulated GC content and positively regulated EC content in ‘Longjing 43’. In ‘Baiye 1 hao’, *CsMYB2* positively regulated anthocyanin content and negatively regulated PA content.

## Discussion

MYB TFs involved in the flavonoid pathway have been identified in various species. For example, in *Arabidopsis*, a number of MYB members, including TT2, are widely involved in the regulation of flavonoid pathways [10]. In *Fagopyrum*, the *FtMYB123L* gene was used as a homologous gene for *TT2* and was found to regulate the flavonoid pathway [17]. To date, the relationship between MYB TFs and the flavonoid pathway in tea plants remains unknown.

In this study, two MYB genes (*CsMYB2* and *CsMYB26*), which are similar to AtMYB123 based on the phylogenetic tree, were identified based on the transcriptome of tea plant [3]. Multiple sequence alignment showed two conserved SANT domains: motif1 and motif2. Motif1 was in contact with the bHLH TF. To date, the function of motif2 is unknown [15]. Phylogenetic analysis suggested that CsMYB2 and CsMYB26 have marked orthologous relationships with VvMYBPAR and FcMYB251, respectively. The CsMYB2 and CsMYB26 TFs were grouped into the PA biosynthesis-related MYB clade. The *TT2* gene of *A. thaliana* was found to localize in the nucleus through subcellular localization assays [10]. This finding was consistent with the experimental results of this study, which showed that the *CsMYB2* gene also localized in the nucleus.

Our study showed that catechins were mainly detected in old leaves and were almost undetectable in young leaves and mature leaves. EC was mainly found in tender leaves, and its content in old leaves and mature leaves was low [27]. The expression profiles of *CsDFR* and *CsANR* were positively correlated with PA content in ‘Longjing 43’; however, no such correlation was found in ‘Baiye 1 hao’. The biosynthesis and degradation processes involved in the polymerization, esterification, and carbonylation reactions in the flavonoid pathway are very complex and are subject to dynamically changing regulation in the context [28].

**Fig. 13:**
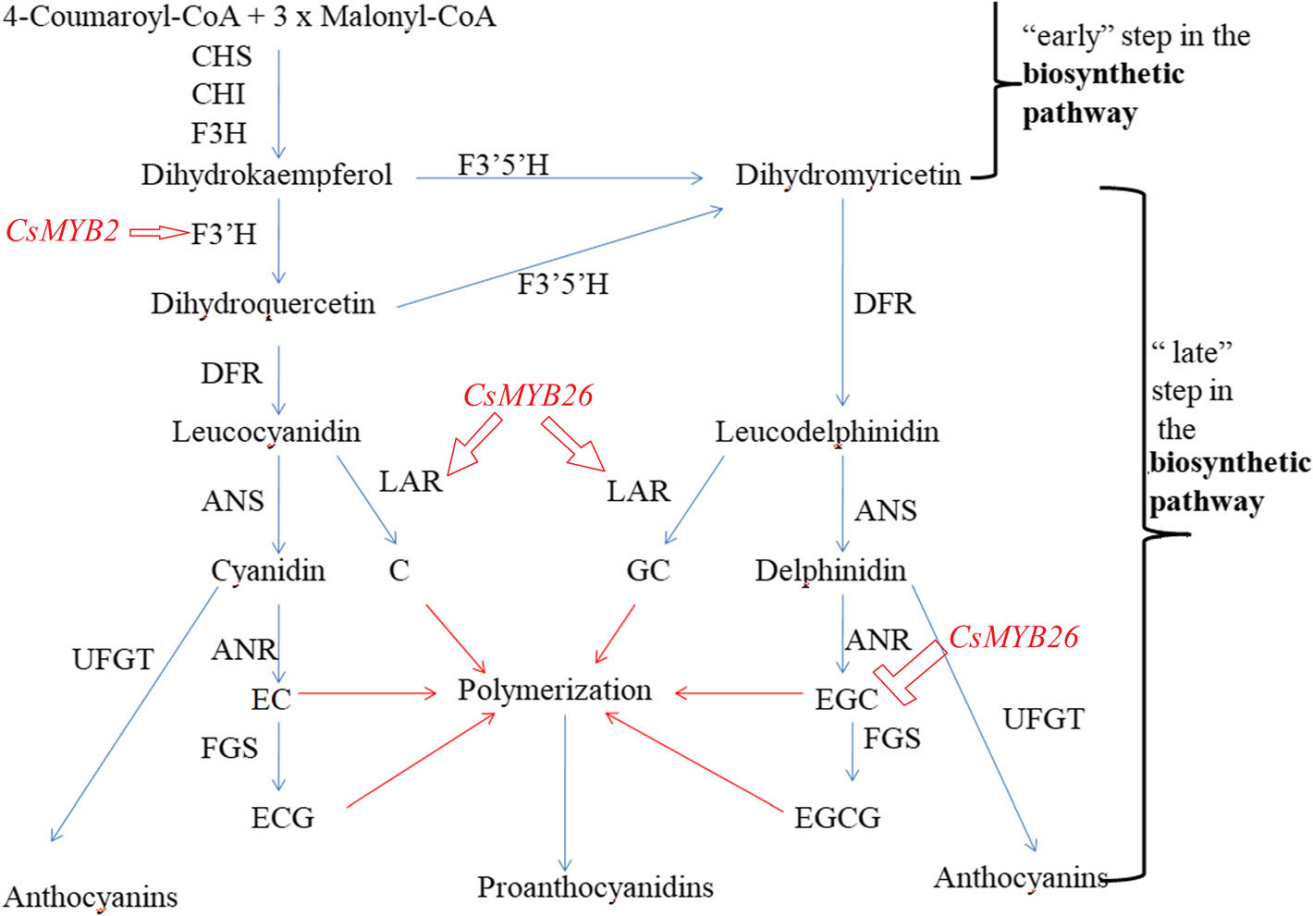
Content analysis of anthocyanins and soluble proanthocyanidins. **a** Anthocyanin content. **b** Soluble proanthocyanidin content

According to gene association analysis, a probable regulatory network in *CsMYB2* and *CsMYB26* is involved in the flavonoid pathway (Fig. 13). The results of the present study suggested that *CsMYB2* positively regulated *CsF3’H* in the two tea plant cultivars and that *CsMYB26* expression was positively correlated with *CsLAR* expression and negatively correlated with EGC content in both tea plant cultivars. This observation was related to the MYB TF levels. Nakatsuka et al. isolated and identified MYB5a and MYB5b, which independently regulate the early steps of flavonoid biosynthesis, resulting in an increase in flavonoid products [29]. As a homologous gene with *CsMYB26* and *CsMYB2*, *LjTT2*, when overexpressed, increased the expression levels of *DFR* and *ANS* and the contents of anthocyanins and PAs in *A. thaliana*. LjTT2 can directly regulate the late flavonoid pathway [30]. Passeri et al. transferred the *VvMYBPA1* gene of grapevine into *A. thaliana* and found that the increase in PA content was mainly based on the EC-based oligomer composition [18]. Thus, MYB TFs play important roles in the flavonoid pathway.

Promoter analysis indicated that *CsMYB2*, *CsMYB26* and the related structural genes (*CsF3’H*, *CsDFR*, *CsANS*, *CsANR*, *CsLAR*, and *CsUFGT*) contain several MYB recognition elements. The expression of the structural genes might be regulated by *CsMYB* genes, and the expression of *CsMYB2* and *CsMYB26* might also be regulated by other *CsMYB* genes.

These genes also contain several light- and ABA-responsive elements. The expression of these genes was downregulated under ABA treatment, indicating that ABA may reduce the expression of structural genes in the flavonoid pathway by regulating the *CsMYB2* and *CsMYB26* genes, leading to a decrease in flavonoid metabolites. The expression levels of most genes were lower under shading treatment than under sunlight treatment, indicating that *CsMYB2* and *CsMYB26* may regulate and reduce the expression of structural genes in the flavonoid pathway, leading to a decrease in flavonoid metabolites.

## Conclusion

In this study, the content of secondary metabolites in the flavonoid pathway were detected in two tea plant cultivars. The correlation of the expression levels of *CsMYB2* and *CsMYB2*6, two R2R3-MYB TFs, and some structural genes involved in the flavonoid biosynthesis pathway were also analysed. The regulatory mechanism governing *CsMYB2* and *CsMYB26* involvement in the flavonoid biosynthesis pathway was evaluated and predicted. Our findings suggested that *CsF3’H* expression might be controlled by *CsMYB2* and that *CsLAR* expression might be regulated by *CsMYB26*. The results also demonstrated that the EGC content was intimately linked to *CsMYB26* expression with a negative correlation. Moreover, *CsMYB2* and *CsMYB26* were confirmed to be involved in the flavonoid biosynthesis pathway. Future studies should explore the possibility of improving flavonoid metabolism via transgenic engineering in tea plant leaves.

## Material and methods

### Preparation of plant materials

**Fig. 14:**
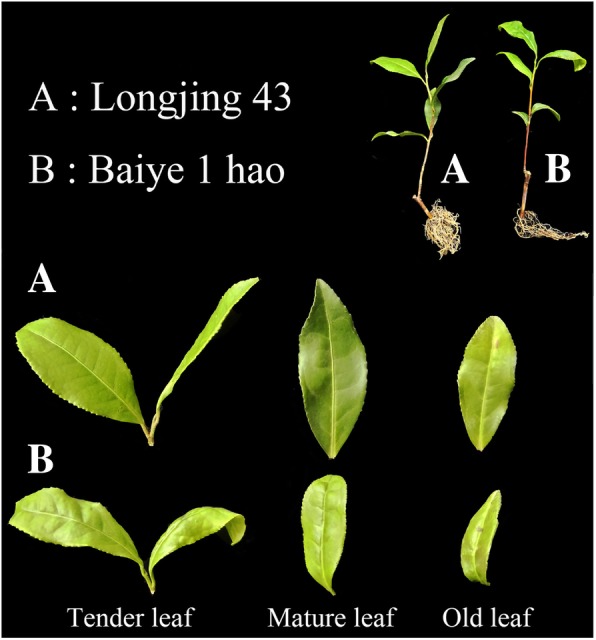
A possible functional network of the flavonoid biosynthetic pathway and associated regulated genes in tea plant

The two tea plant cultivars [*Camellia sinensis* (L.) O. Kuntze cv. ‘Longjing 43’ and ‘Baiye 1 hao’] were deposited in Tea Science Research Institute of Nanjing Agricultural University (Nanjing, China). The 1-year-old vegetatively propagated cuttings of the tea plant cultivars ‘Longjing 43’ and ‘Baiye 1 hao’ (Fig. 14) were planted in a phytotron. The relative humidity was programmed at 70 ± 10%, the temperature was 25 °C, and the light intensity was 300 μmol m^− 2^ s^− 1^, with 16 h of light during the daytime and 8 h of darkness. Samples were collected with a mixture of corresponding tender leaves, mature leaves and old leaves, immediately frozen in liquid nitrogen, and stored at − 80 °C. The tea plant cultivar ‘Longjing 43’ was also used for shading and ABA treatment assays. Shade netting (60% ± 5% light transmission) was used for the shading treatment. The tender leaves of tea plant were harvested at 3, 6, and 9 d after the shading treatment, and tea plants maintained under sunlight conditions were used as controls. In addition, tea plant seedlings were sprayed with 200 μM ABA solution, and the tender leaves were harvested at 0, 2, 4, 8, and 24 h after treatment. All of the collected samples were immediately frozen in liquid nitrogen and stored at − 80 °C for further analysis.

### Identification of MYB genes in tea plant

According to the tea database in our laboratory [3], a total of 119 fragments related to R2R3-MYB-type genes were detected, and 27 genes containing two incomplete structural domains were screened out. Furthermore, a phylogenetic tree was constructed based on all R2R3-MYB-type *A. thaliana* genes and two R2R3-MYB-type TF genes (*CsMYB2* and *CsMYB26*) involved in the flavonoid biosynthesis pathway.

### RNA isolation, cDNA synthesis, and gene cloning

Total RNA was isolated from samples by using a Quick RNA extraction kit (Aidelai, Beijing, China) according to the manufacturer’s protocol. Reverse transcription was performed using a PrimeScript RT reagent kit with gDNA Eraser (TaKaRa, Dalian, China), and the samples were then diluted 15-fold in preparation for quantitative real-time PCR (qRT-PCR).

*CsMYB2* and *CsMYB26* were amplified from the ‘Longjing 43’ cultivar, and the primer sequences are listed in Table 3. The PCR program was as follows: 95 °C for 5 min; followed by 35 cycles at 95 °C for 30 s, 55 °C for 15 s, and 72 °C for 60 s; and 72 °C for 10 min. The PCR products were recovered and inserted into the pMD-19 T plasmid vector (TaKaRa, Dalian, China), and the ligation mixture was transformed into the *E. coli* strain DH5α. Ampicillin resistance was used to identify the clones, which were then sequenced at Tongyong Biotechnology, Inc. (Chuzhou, China).

**Table 3:** Primers used in the experiments

Gene	Direction	Sequence (5′-3′)	Function
*CsMYB2*	Forward	ATGGGGAGAAGCCCTTGTTGCGCG	Full-length clone
	Reverse	CTAGACAAATTGCCAATCCCCTCC	
*CsMYB26*	Forward	ATGGGGAGGAGTCCATGCTGC	Full-length clone
	Reverse	TCATGGCCAGTCCTCAGAATCAAG	
*CsMYB2*	Forward	TTACAGCAATGACAACAA	RT-qPCR
	Reverse	CCAGACTCCAGAATAGAA	
*CsMYB26*	Forward	CTCCAACTCCATAATATCAA	RT-qPCR
	Reverse	GAACAAGGTATCGTCATAA	
*CsDFR*	Forward	TGCAGAGAGAAGGGTTTGCT	RT-qPCR
	Reverse	AAGGCAAGGCACCAATACAC	
*CsANS*	Forward	TCGAGCCCTAGCTACCAAGA	RT-qPCR
	Reverse	CAAGTCAGTGTGGGCTTCAA	
*CsANR*	Forward	GCCTGGTCATGGATGAGAGT	RT-qPCR
	Reverse	GGCCATGAGAGTAGGGATGA	
*CsLAR*	Forward	GGGGCATCCTGTATCAAAGA	RT-qPCR
	Reverse	CCGCATACCTTTCAGTCCAT	
*CsUFGT*	Forward	GCACCATAACCACCCCACC	RT-qPCR
	Reverse	TGTCACAAACACACCAACCGAT	
*CsF3’H*	Forward	CTATTGCAGCTTCTTGATGATCCGA	RT-qPCR
	Reverse	GCTCTTTGGTTGCTTTGTTGATTAG	
*CsMYB2*	Forward	CACCATCACCATCACGCCATGATGGGGAGAAGCCCTTGTTGCGCG	Subcellular localization
	Reverse	CACTAGTACGTCGACCATGGCGACAAATTGCCAATCCCCTCC	
*GAPDH*	Forward	TTGGCATCGTTGAGGGTCT	Reference gene
	Reverse	CAGTGGGAACACGGAAAGC	

### Multiple sequence alignment, phylogenetic analysis, and conserved motif analysis

The sequences of nucleotides and amino acids were analysed with BLAST on the NCBI website (https://blast.ncbi.nlm.nih.gov/Blast.cgi). The sequences of AtR2R3-MYB family TFs were downloaded from the PlantTFDB website (http://planttfdb.cbi.pku.edu.cn/) [31]. DNAMAN version 6.0 was utilized to analyse the full alignment. MEGA5 was used to generate the molecular phylogenetic tree with the neighbor-joining method [32].

### Analysis of promoter regions and interaction networks of CsMYB2, CsMYB26 and the structural genes involved in the flavonoid pathway

The functional interaction networks of CsMYB2, CsMYB26 and the structural genes involved in the flavonoid pathway were constructed using STRING software. The sequences 2000 bp upstream of the transcription start site, as promoter regions, were collected from the tea plant genome database (http://pcsb.ahau.edu.cn:8080/CSS/) [33] and submitted to the PlantCARE database (http://bioinformatics.psb.ugent.be/webtools/plantcare/html/) [34] to search for putative *cis*-acting elements.

### Subcellular localization analysis of CsMYB2

Subcellular localization analysis was mainly performed according to Li et al.’s method with some modification [35]. Briefly, the *CsMYB2* gene was amplified with the stop codons deleted and was inserted into the pA7 vector. The pA7 vector was utilized as a control expressing a 35S::GFP (green fluorescent protein) fusion protein. The constructed plasmids were transferred into onion (*Allium cepa*) epidermal cells by a helium-driven particle accelerator (PDS-1000, Bio-Rad) and were then incubated for 16 h at 22 °C on 1/2 Murashige and Skoog (1/2MS) medium in the dark. The GFP signals were observed using an LSM 780 confocal microscopy imaging system (Zeiss, Germany).

### Gene expression level analysis

A Bio-Rad iQ5 fluorescence quantitative PCR platform was used to perform qRT-PCR with SYBR Premix Ex Taq (TaKaRa, Dalian, China). The experiment was performed in a volume of 20 μL: 10 μL SYBR premix, 0.4 μL each specific primer, 2 μL diluted cDNA, and 7.2 μL ddH_2_O. The cycling conditions for qRT-PCR were as follows: 95 °C for 3 min, followed by 40 cycles at 95 °C for 10 s and 60 °C for 20 s. All reactions were performed in triplicate. *GAPDH* was utilized for normalization of the expression levels [27], and the 2^−ΔΔCt^ method was used to calculate the relative gene expression levels [36]. The primers used in this work are listed in Table 3.

### Determination of catechin content from tea plant leaves

To determine the concentrations of catechin monomers, samples were prepared using the acidified methanol method described by Wu et al. [3]. Briefly, samples of approximately 0.2 g dry weight were extracted in 5 mL acetonitrile/water (75:25). The samples were sonicated at room temperature for 10 min and centrifuged for 10 min at 5000 rpm, and the residues were re-extracted as described above. The collected volume was raised to a total volume of 10 mL. Then, 2 mL extract was added to 8 mL of the extract-stabilized solution. Finally, the membrane was filtered through a 0.22 μm organic membrane and analysed with HPLC. A 5 μL sample was used for reversed-phase HPLC analysis.

### Determination of anthocyanin content in tea plant leaves

The anthocyanin extraction protocol was modified based on Pang et al.’s method [37]. Briefly, approximately 0.5 g mixed tissue samples was ground under liquid nitrogen, extracted with 5 mL methanol (0.1% HCl) for 1 h with ultrasound, and then centrifuged for 10 min at 5000 rpm. The supernatant was collected, 2 mL water and 2 mL chloroform were added to 2 mL extract to remove chlorophyll, and the sample was then centrifuged for 10 min at 5000 rpm. The supernatant was collected and filtered through a 0.45 μm organic membrane, and the absorption of the aqueous phase was measured at 530 nm and 600 nm. The total anthocyanin content was calculated based on the molar absorbance of cyanidin-3-*O*-glucoside.

### Determination of PA content from tea plant leaves

As were extracted based on the method of Jiang et al. [38]. Briefly, approximately 0.5 g mixed tissue samples were ground under liquid nitrogen, extracted with 3 mL 70% acetone/0.5% acetic acid (extraction solution) by vortexing, and centrifuged for 10 min at 5000 rpm. The residues were re-extracted twice as described above, and the collected volume was raised to a total volume of 10 mL. Then, 2 mL water and 2 mL chloroform were added to 2 mL extract to remove chlorophyll, and the sample was centrifuged for 10 min at 5000 rpm. For the quantification of soluble PAs, 0.5 mL supernatant was added to a 95% butanol-HCl mixture. The mixtures were sonicated and boiled for 1 h and were then cooled to room temperature, followed by centrifugation at 5000 rpm for 10 min. The supernatant was collected and filtered through a 0.45 μm organic membrane. The absorbances at 550 nm and 600 nm were measured. Cyanidin was used as a standard for the calibration curve. Three technical replicates were performed.

### Statistical analyses

A Pearson correlation coefficient (PCC) analysis was performed to evaluate the correlations between *CsMYB2* and *CsMYB26* expression levels, the expression levels of 6 structural genes in the flavonoid pathway and the contents of catechin, anthocyanins and soluble PA using SPSS software. *P*-values less than 0.05 were considered statistically significant.

## Additional file

Additional file 1: Putative cis-acting regulatory elements of CsMYB2, CsMYB26 and the structural genes involved in the flavonoid pathway. (XLS 33 kb)

## Abbreviations

ANR: Anthocyanidin reductase
ANS: Anthocyanidin synthase
C: Catechin
CHI: Chalcone isomerase
CHS: Chalcone synthase
DFR: Dihydroflavonol 4-reductase
EC: Epicatechin
ECG: Epicatechin gallate
EGC: Epigallocatechin
EGCG: Epigallocatechin gallate
F3’5’H: Flavanone 3′,5′-hydroxylase
F3’H: Flavonoid 3′ hydroxylase
F3H: Flavanone 3-hydroxylase
GC: Gallocatechin
GFP: Green fluorescent protein
LAR: Leucoanthocyanidin reductase
NJ: Neighbor-joining
ORF: Open reading frame;
PA: Proanthocyanidin
PCC: Pearson correlation coefficient
qRT-PCR: Quantitative real-time polymerase chain reaction
RP-HPLC: Reversed-phase high-performance liquid chromatography
TF: Transcription factor
UFGT: UDP-glucose: flavonoid 3-O-glucosyltransferase
1/2MS: 1/2 Murashige and Skoog
