# Correction to: Two MYB transcription factors (CsMYB2 and CsMYB26) are involved in flavonoid biosynthesis in tea plant [*Camellia sinensis* (L.) O. Kuntze]

**DOI:** 10.1186/s12870-018-1582-0

**Published:** 2019-01-21

**Authors:** Wen-Li Wang, Yong-Xin Wang, Hui Li, Zhi-Wei Liu, Xin Cui, Jing Zhuang

**Affiliations:** Tea Science Research Institute, College of Horticulture, Nanjing Agricultural University, 1 Weigang, Nanjing, 210095 Jiangsu China

**Correction to: Wang et al. BMC Plant Biology**

**https://doi.org/10.1186/s12870-018-1502-3**

Following publication of the original article [1], the author reported that there was a mismatch between figures and their legends. The correct figures and legends are as follows:

**Correction 1: Page 3 (**Fig. 1**).**

**Fig. 1:**
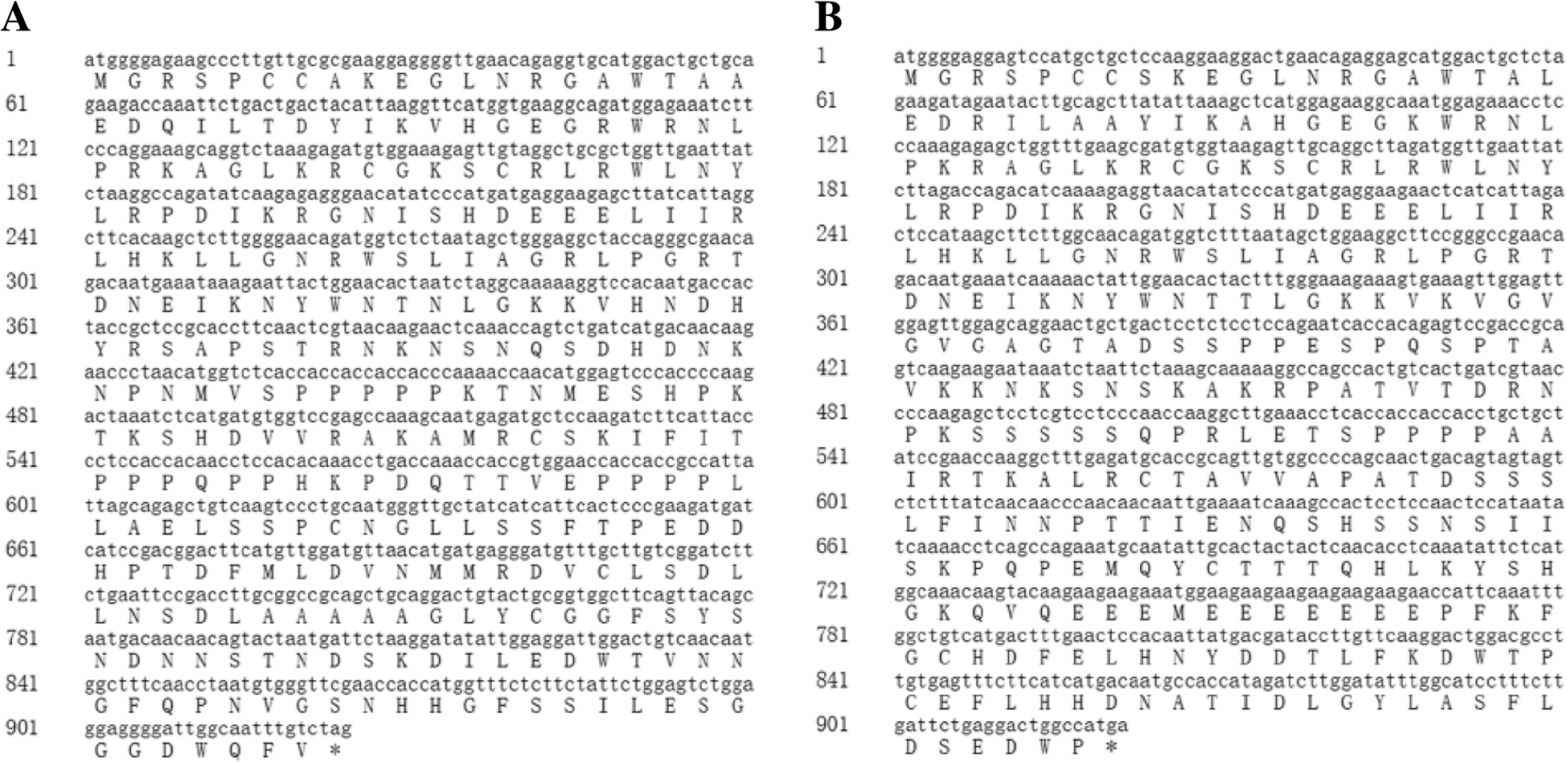
Gene sequences of *CsMYB2* and *CsMYB26* with the deduced amino acid sequences. **a** *CsMYB2* gene. **b** *CsMYB26* gene

Please change the legend to ‘Fig. 1 Gene sequences of *CsMYB2* and *CsMYB26* with the deduced amino acid sequences. (A) *CsMYB2* gene. (B) *CsMYB26* gene*.*’.

**Correction 2: Page 4 (**Fig. 2**).**

**Fig. 2:**
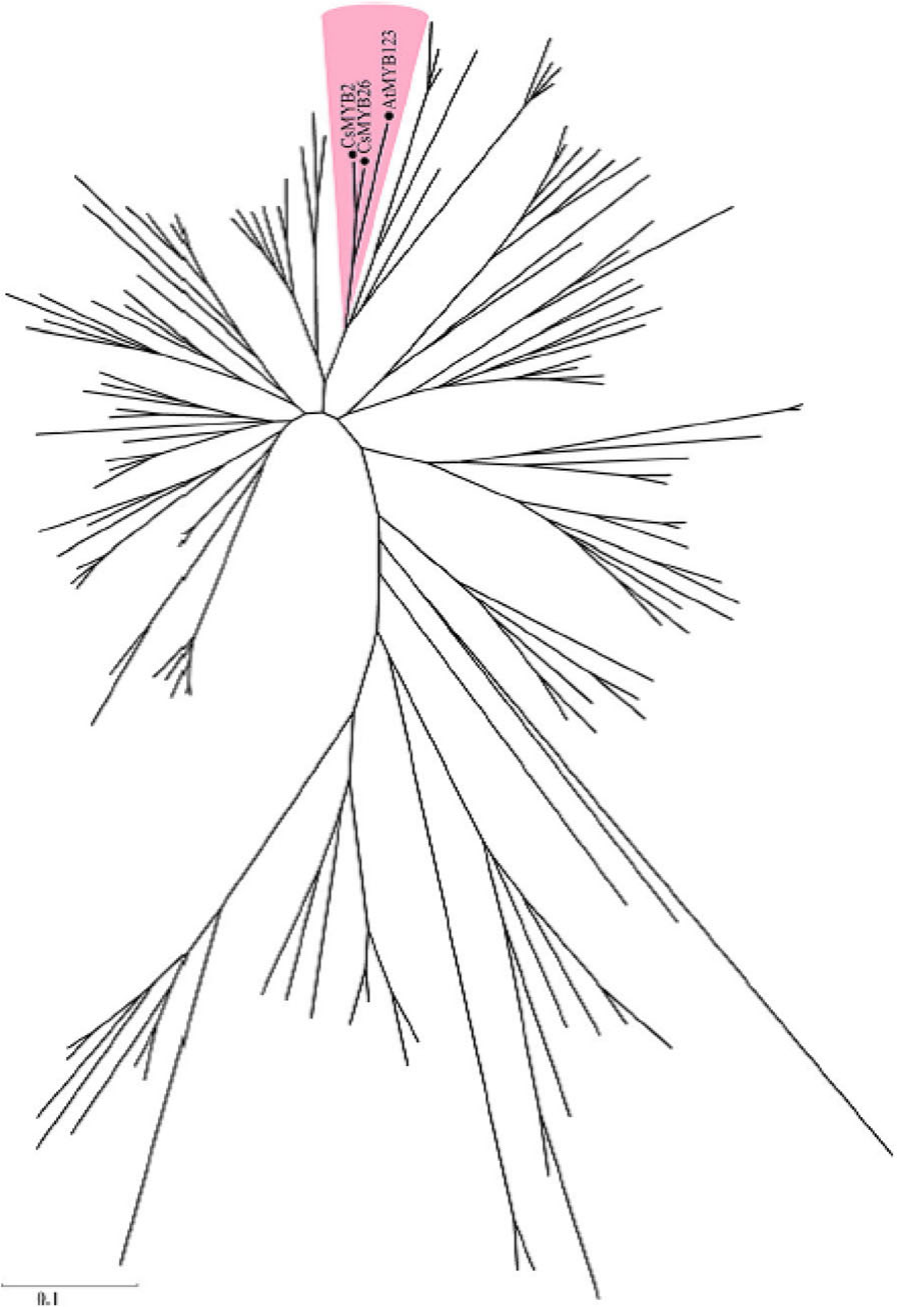
Unrooted phylogenetic tree of CsMYB2 and CsMYB26 with R2R3-MYB-type *A. thaliana* TFs. A phylogenetic tree was built using the neighbor-joining method with MEGA 5 software. The putative functions of all R2R3-MYBs are listed on the right

Please change the legend to ‘Fig. 2 Unrooted phylogenetic tree of CsMYB2 and CsMYB26 with R2R3-MYB-type *A. thaliana* TFs. A phylogenetic tree was built using the neighbor-joining method with MEGA 5 software. The putative functions of all R2R3-MYBs are listed on the right.’.

**Correction 3: Page 5 (**Fig. 3**).**

**Fig. 3:**
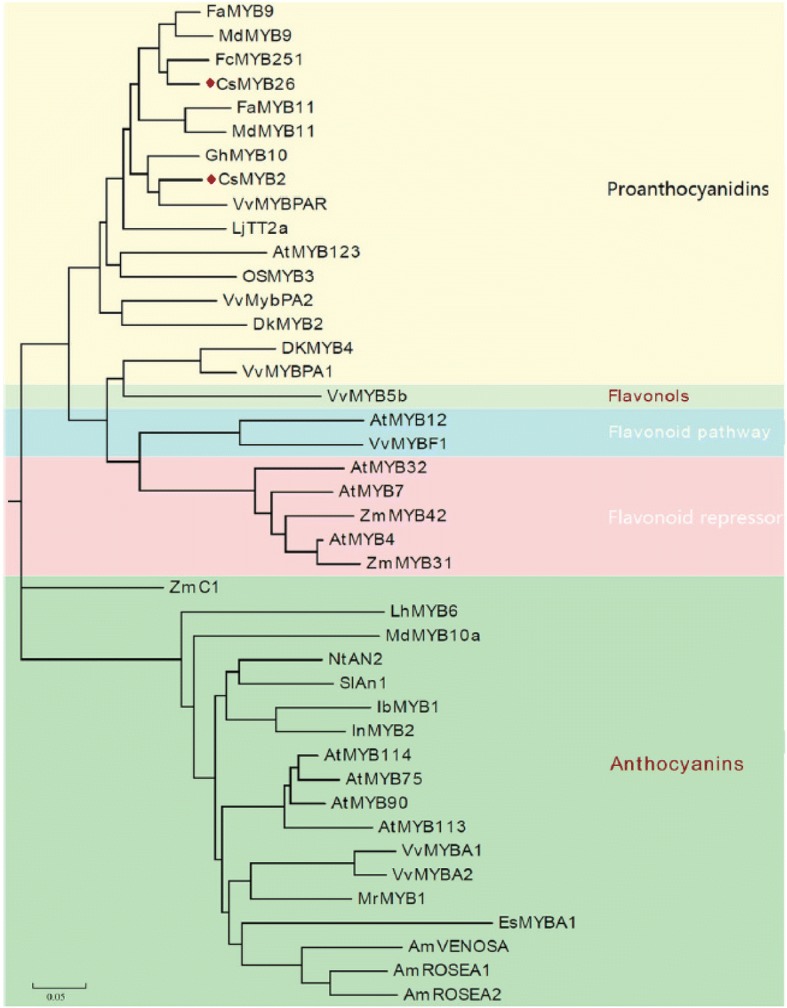
Phylogenetic relationships among CsMYB2, CsMYB26 and flavonoid-related R2R3-MYBs from other plant species. A phylogenetic tree was built using the neighbor-joining method with MEGA 5 software. The putative functions of all R2R3-MYBs are listed on the right

Please change the legend to ‘Fig. 3 Phylogenetic relationships among CsMYB2, CsMYB26 and flavonoid-related R2R3-MYBs from other plant species.

A phylogenetic tree was built using the neighbor-joining method with MEGA 5 software. The putative functions of all R2R3-MYBs are listed on the right.’.

**Correction 4: Page 6 (**Fig. 4**).**

**Fig. 4:**
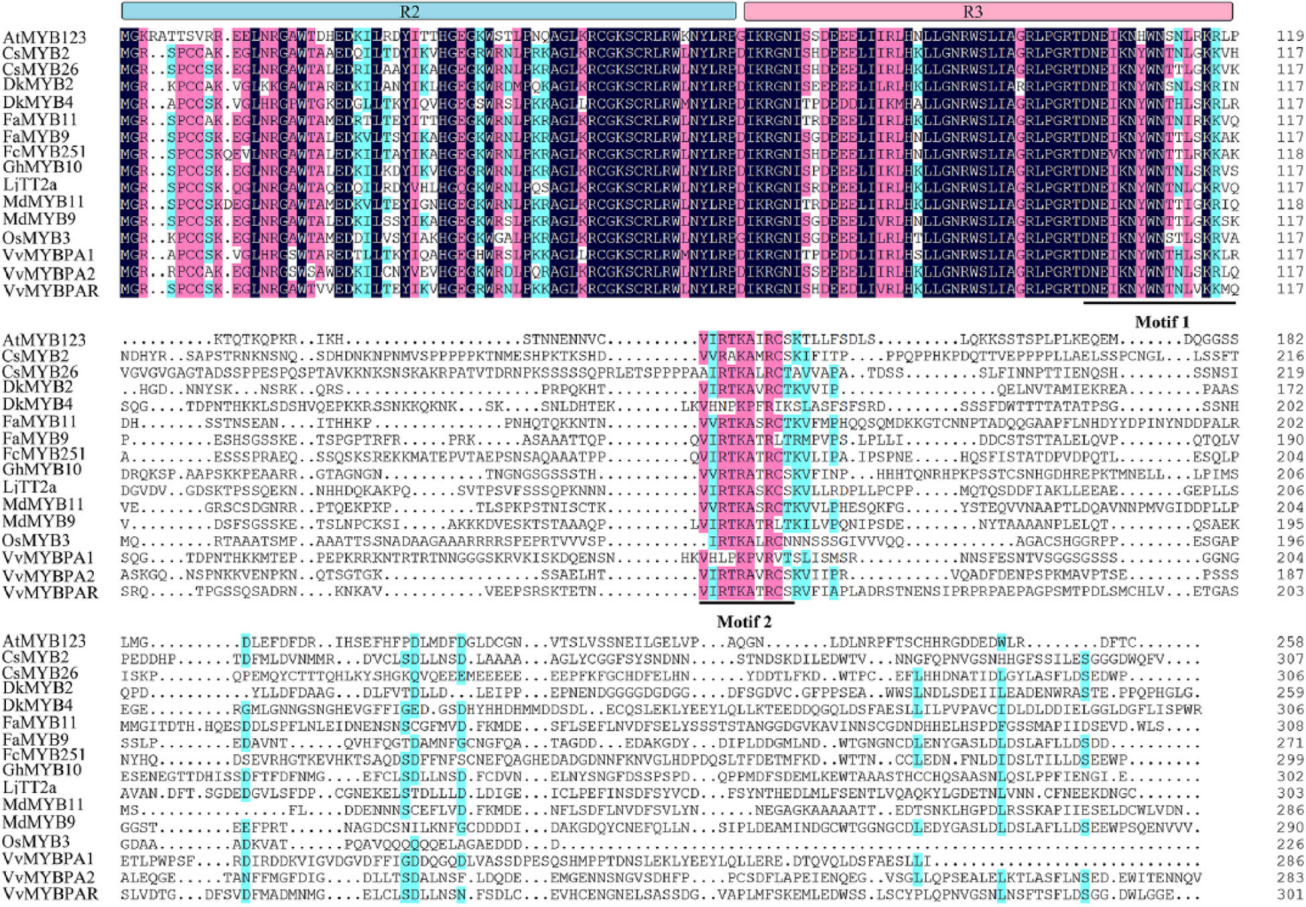
Alignment of the deduced amino acid sequences of CsMYB2 and CsMYB26 with those of R2R3-MYB proteins from other plant species

Please change the legend to ‘Fig. 4 Alignment of the deduced amino acid sequences of CsMYB2 and CsMYB26 with those of R2R3-MYB proteins from other plant species.’.

**Correction 5: Page 6 (**Fig. 5**).**

**Fig. 5:**
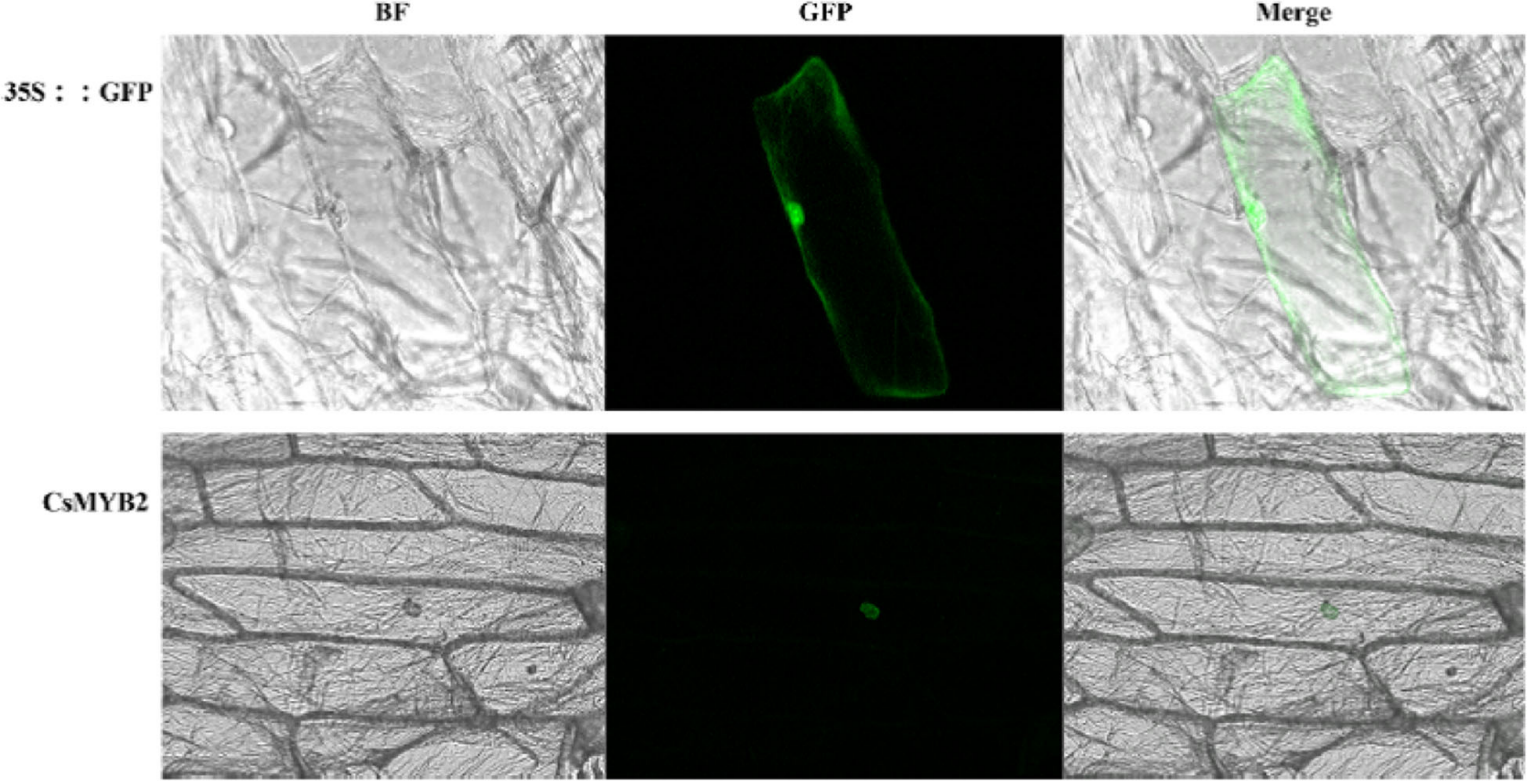
Subcellular localization of CsMYB2. BF: Bright-field microscopy image. GFP: Green fluorescence image. Merge: Merged bright-field and green fluorescence images

Please change the legend to ‘Fig. 5 Subcellular localization of CsMYB2.

BF: Bright-field microscopy image. GFP: Green fluorescence image. Merge: Merged bright-field and green fluorescence images.’

**Correction 6: Page 7 (**Fig. 6**).**

**Fig. 6:**
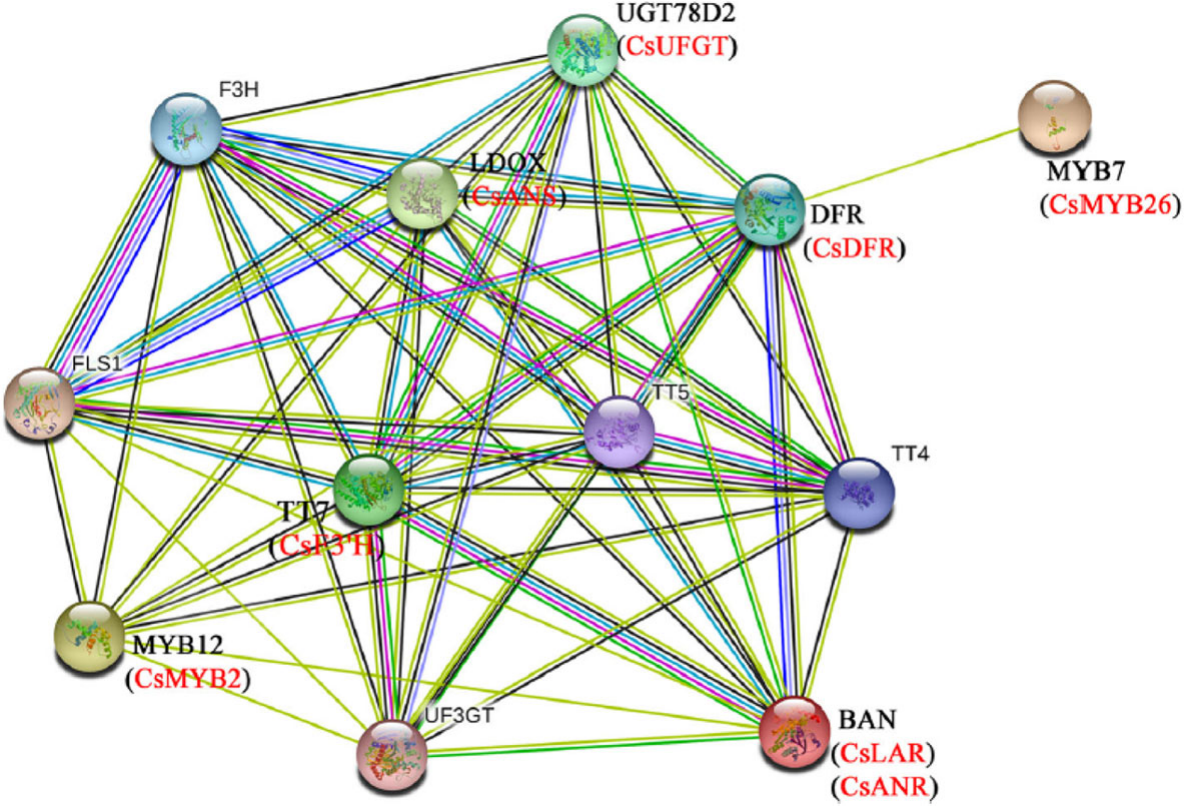
Interaction network of CsMYB2, CsMYB26 and the structural genes involved in flavonoid biosynthesis

Please change the legend to ‘Fig. 6 Interaction network of CsMYB2, CsMYB26 and the structural genes involved in flavonoid biosynthesis.’.

**Correction 7: Page 8 (**Fig. 7**).**

**Fig. 7:**
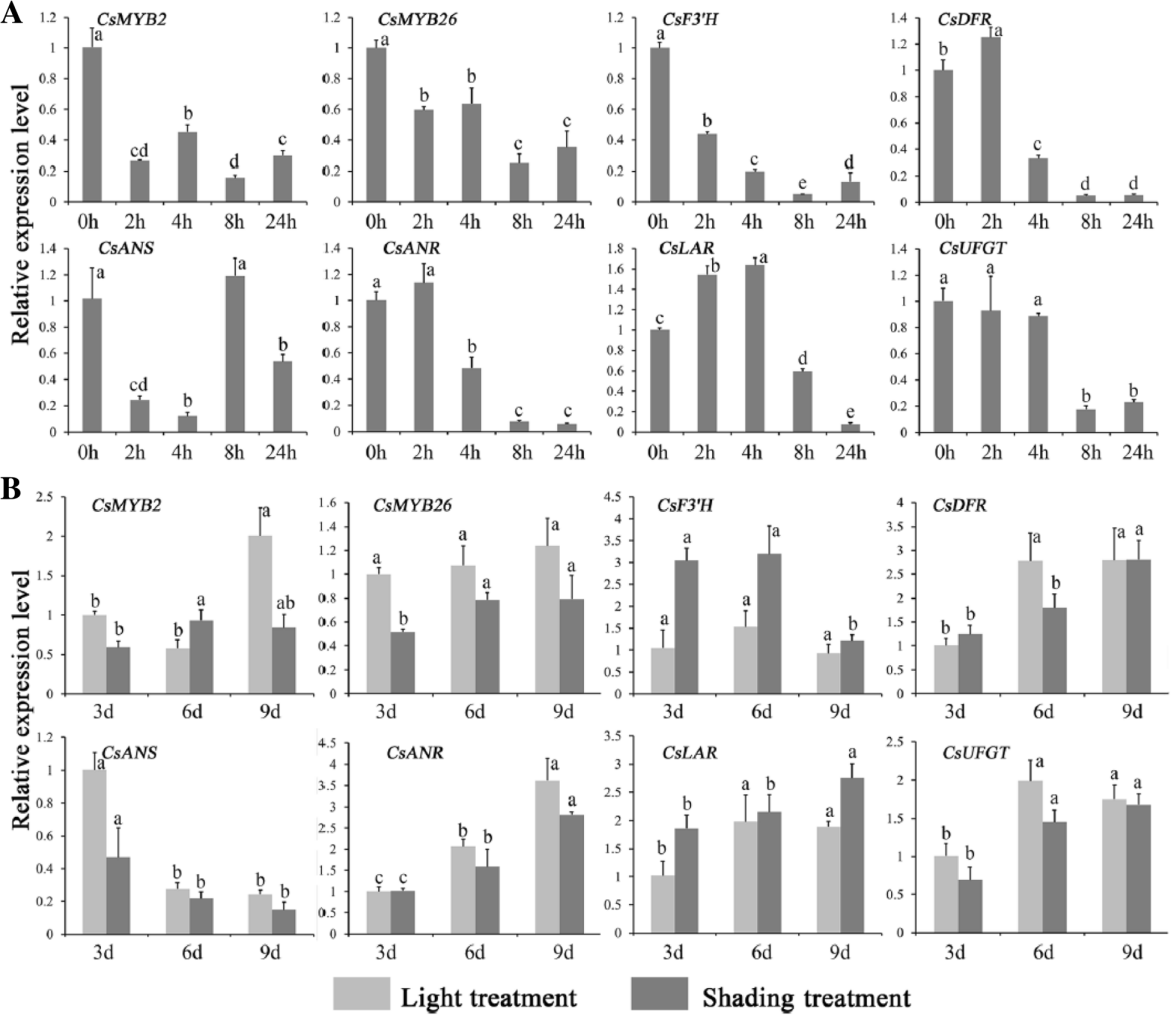
Expression profiles of *CsMYB2*, *CsMYB26* and structural genes under ABA and shading treatments. **a** ABA treatment. **b** shading treatment

Please change the legend to ‘Fig. 7 Expression profiles of *CsMYB2*, *CsMYB26* and structural genes under ABA and shading treatments. (A) ABA treatment. (B) shading treatment.’.

**Correction 8: Page 8 (**Fig. 8**).**

**Fig. 8:**
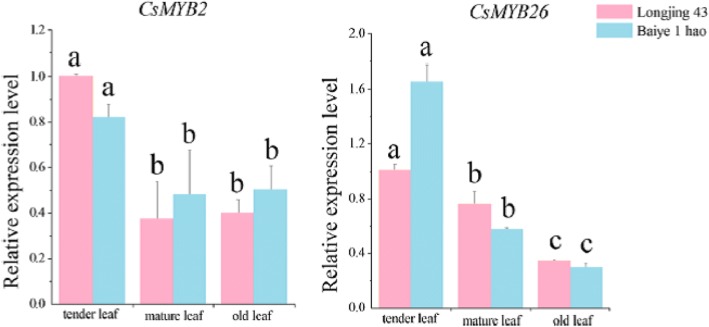
Relative expression analyses of *CsMYB2* and *CsMYB26* in the leaves from different sites in tea plant

Please change the legend to ‘Fig. 8 Relative expression analyses of *CsMYB2* and *CsMYB26* in the leaves from different sites in tea plant.’.

**Correction 9: Page 9 (**Fig. 9**).**

**Fig. 9:**
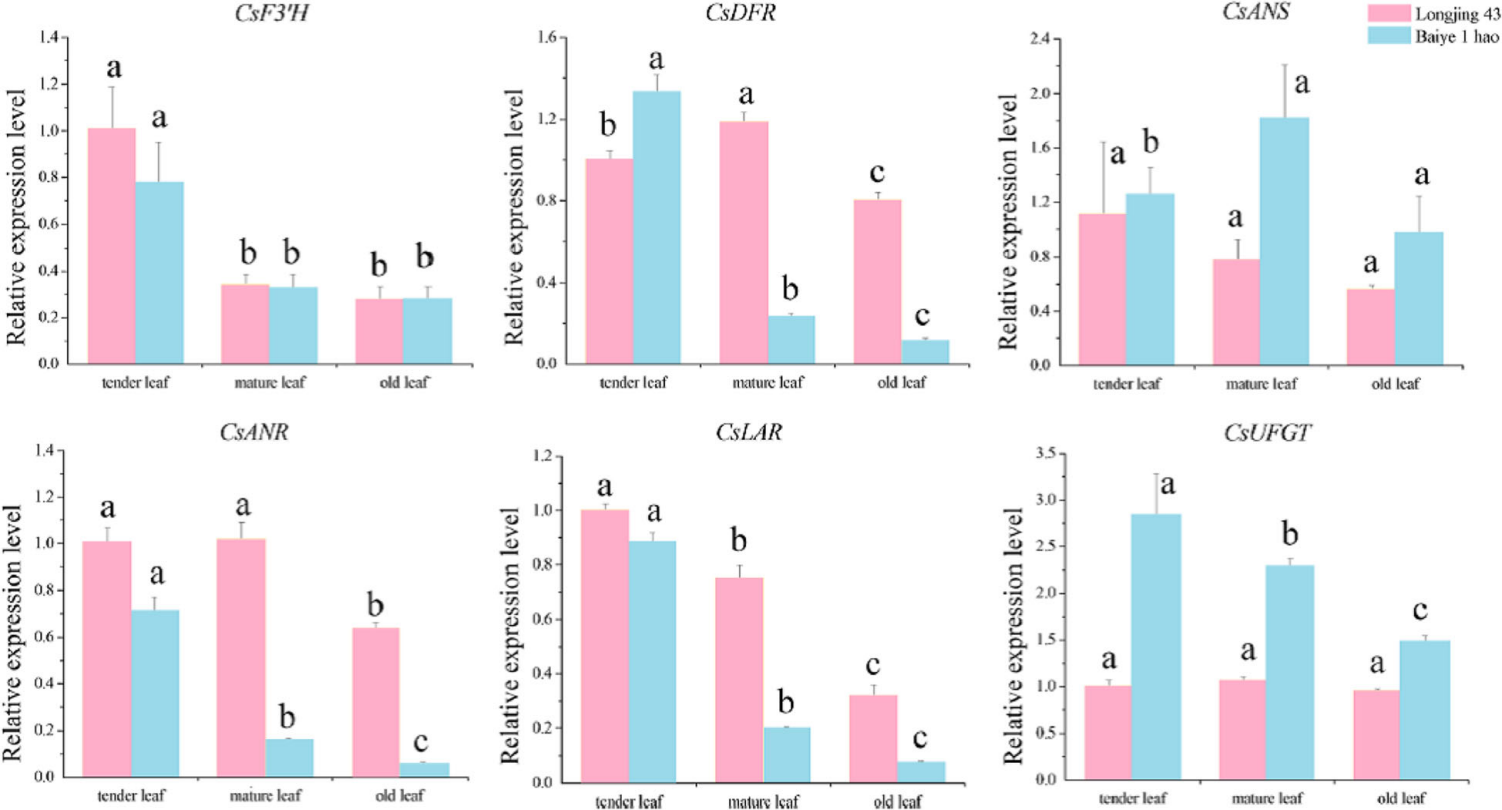
Relative expression analyses of genes involved in the flavonoid biosynthesis pathway in the leaves from different sites in tea plant

Please change the legend to ‘Fig. 9 Relative expression analyses of genes involved in the flavonoid biosynthesis pathway in the leaves from different sites in tea plant.’.

**Correction 10: Page 9 (**Fig. 10**).**

**Fig. 10:**
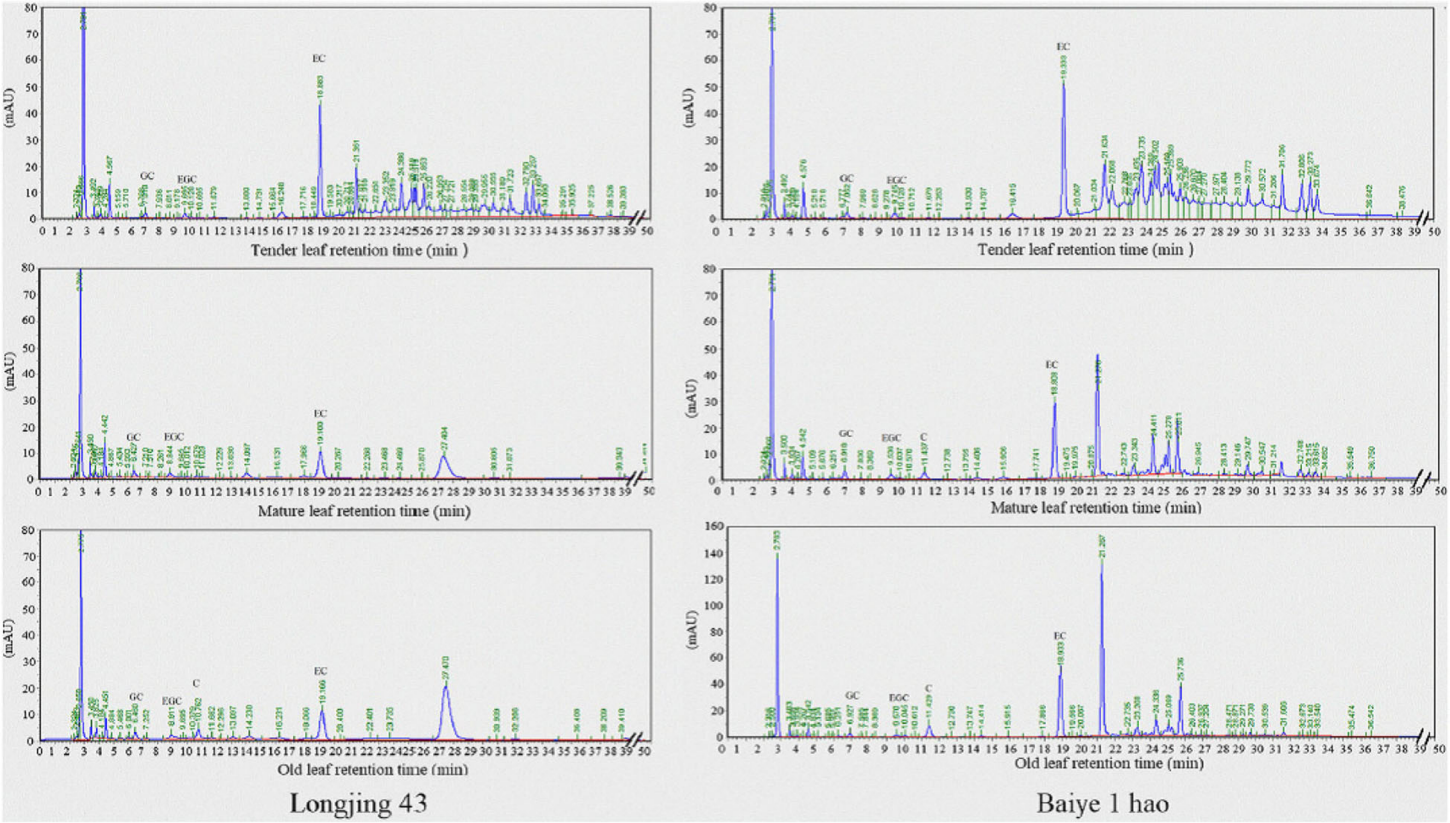
HPLC chromatogram of catechins in leaves from different sites in the ‘Longjing 43’ and ‘Baiye 1 hao’ cultivars

Please change the legend to ‘Fig. 10 HPLC chromatogram of catechins in leaves from different sites in the ‘Longjing 43′ and ‘Baiye 1 hao’ cultivars.’.

**Correction 11: Page 10 (**Fig. 11**).**

**Fig. 11:**
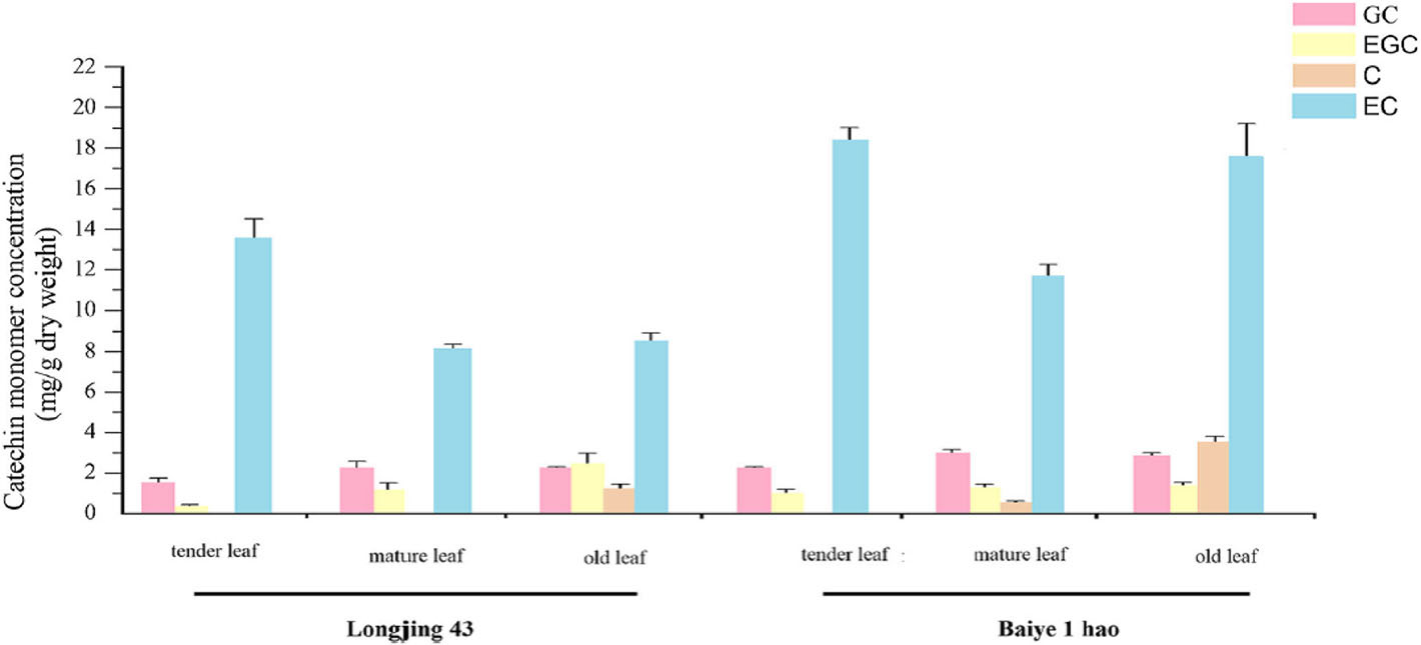
Various catechin monomer content analyses were performed

Please change the legend to ‘Fig. 11 Various catechin monomer content analyses were performed.’.

**Correction 12: Page 10 (**Fig. 12**).**

**Fig. 12:**
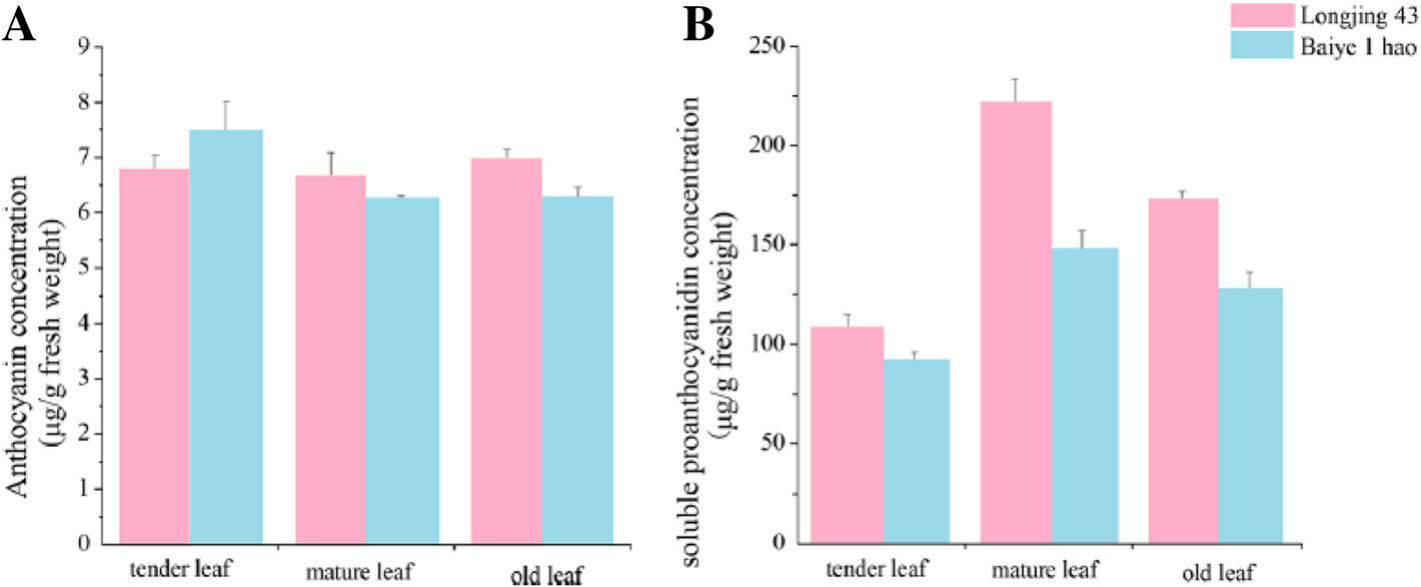
Content analysis of anthocyanins and soluble proanthocyanidins. **a** Anthocyanin content. **b** Soluble proanthocyanidin content

Please change the legend to ‘Fig. 12 Content analysis of anthocyanins and soluble proanthocyanidins. (A) Anthocyanin content. (B) Soluble proanthocyanidin content.’.

**Correction 13: Page 12 (**Fig. 13**).**

**Fig. 13:**
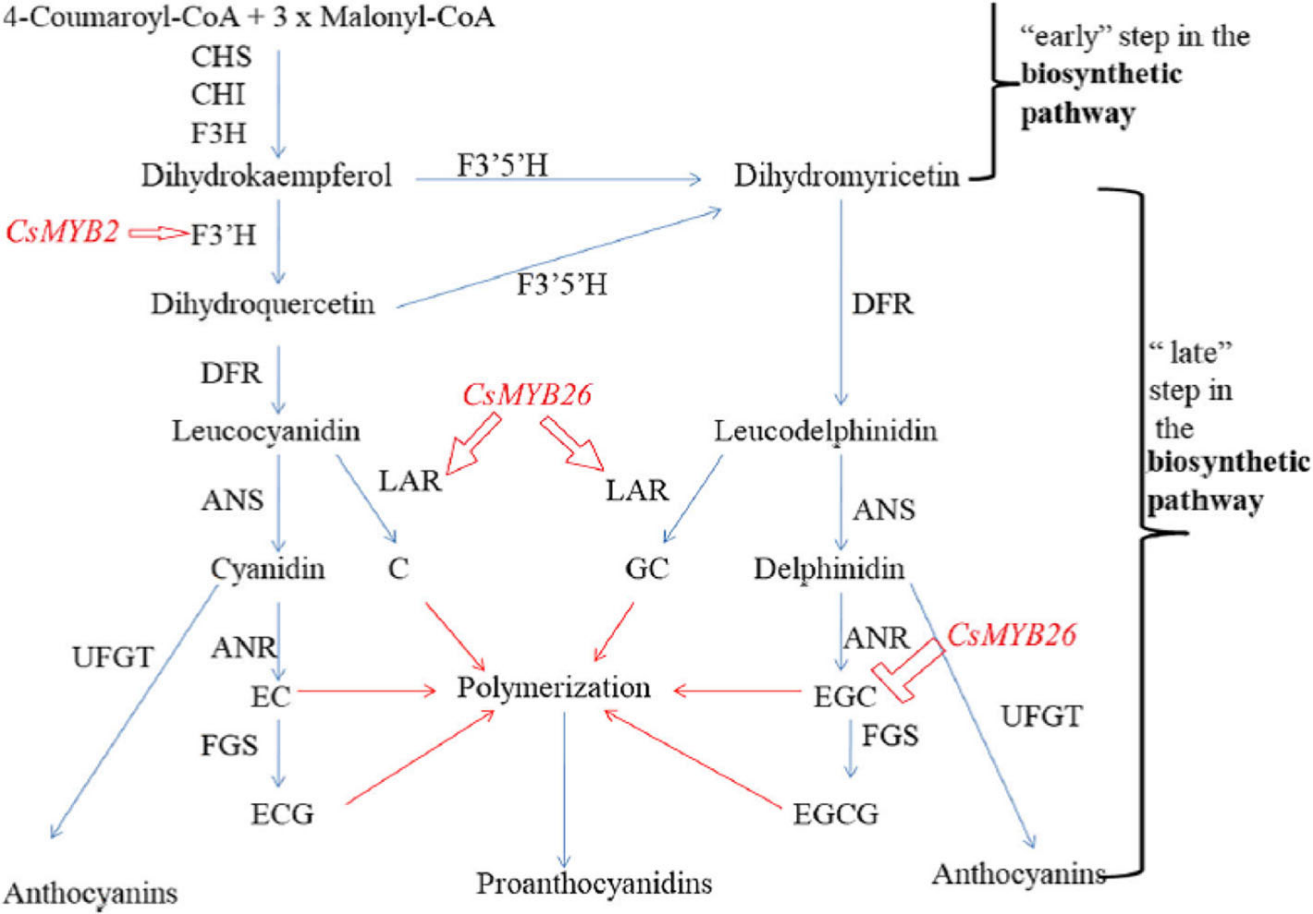
A possible functional network of the flavonoid biosynthetic pathway and associated regulated genes in tea plant

Please change the legend to ‘Fig. 13 A possible functional network of the flavonoid biosynthetic pathway and associated regulated genes in tea plant.’.

**Correction 14: Page 12 (**Fig. 14**).**

**Fig. 14:**
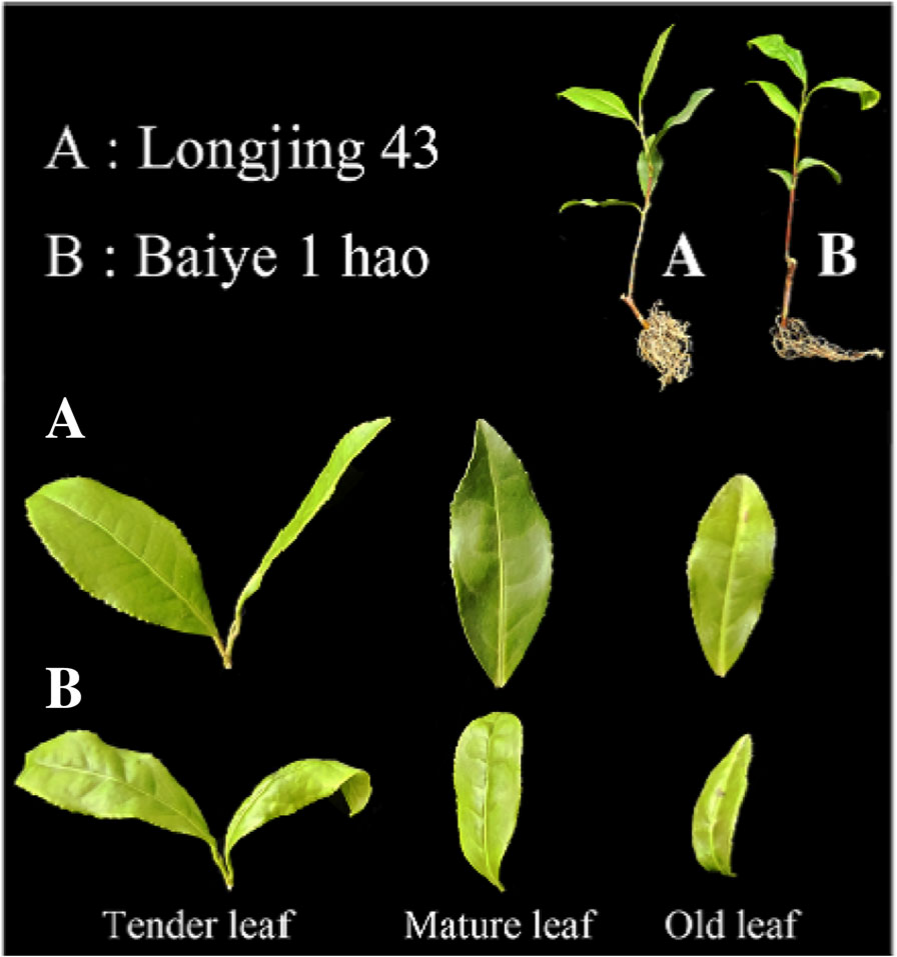
The two tea plant cultivars. **a** ‘Longjing 43’ plant. **b** ‘Baiye 1 hao’ plant

Please change the legend to ‘Fig. 14 The two tea plant cultivars. (A) ‘Longjing 43′ plant. (B) ‘Baiye 1 hao’ plant..’

The original article has been corrected.
